# Small-molecule Akt-activation in airway cells induces NO production and reduces IL-8 transcription through Nrf-2

**DOI:** 10.1186/s12931-021-01865-y

**Published:** 2021-10-19

**Authors:** Indiwari Gopallawa, Li Eon Kuek, Nithin D. Adappa, James N. Palmer, Robert J. Lee

**Affiliations:** 1 Department of Otorhinolaryngology, University of Pennsylvania, Philadelphia, PA 19104 USA; 2 Department of Physiology, Perelman School of Medicine, University of Pennsylvania, Philadelphia, PA 19104 USA

**Keywords:** Antioxidants, Innate immunity, Lung, Oxidative stress, *Pseudomonas aeruginosa*, Toll-like receptor

## Abstract

**Background:**

The non-cancerous functions of Akt in the airway are understudied. In some tissues, Akt phosphorylates and activates endothelial nitric oxide synthase (eNOS) to produce nitric oxide (NO) that has anti-inflammatory effects. NO production has antibacterial and antiviral effects in the airway, and increasing NO may be a useful anti-pathogen strategy. Akt also stimulates the nuclear factor erythroid 2–related factor 2 (Nrf-2) transcription factor, which transcribes antioxidant genes. Therefore, we hypothesized that activation of the Akt/eNOS pathway, which also activates Nrf-2, may have protective effects in human airway cells against injury.

**Methods:**

To directly test the effects of Akt signaling in the airway, we treated A549 and 16HBE cells as well as primary bronchial, nasal, and type II alveolar epithelial cells with small molecule Akt activator SC79. We examined the effects of SC79 on eNOS activation, NO production, Nrf-2 target levels, and interleukin-8 (IL-8) transcription during exposure to TNF-α or *Pseudomonas* flagellin (TLR5 agonist). Additionally, air–liquid interface bronchial cultures were treated with cadmium, an oxidative stressor that causes airway barrier breakdown.

**Results:**

SC79 induced a ~ twofold induction of p-eNOS and Nrf-2 protein levels blocked by PI3K inhibitor LY294002. Live cell imaging revealed SC79 increased acute NO production. Quantitative RT-PCR showed a ~ twofold increase in Nrf-2 target gene transcription. TNF-α or flagellin-induced IL-8 levels were also significantly reduced with SC79 treatment. Moreover, the transepithelial electrical resistance decrease observed with cadmium was ameliorated by SC79, likely by an acute increase in tight junction protein ZO-1 levels.

**Conclusions:**

Together, the data presented here demonstrate SC79 activation of Akt induces potentially anti-pathogenic NO production, antioxidant gene transcription, reduces IL-8 transcription, and may protect against oxidative barrier dysfunction in a wide range of airway epithelial cells.

**Supplementary Information:**

The online version contains supplementary material available at 10.1186/s12931-021-01865-y.

## Background

Obstructive airway diseases are characterized by increased cytokine production, oxidative stress, epithelial remodeling, and loss of epithelial barrier integrity [1, 2]. There is a need for new therapeutic strategies to reduce inflammation and restore barrier function in diseases such as asthma, chronic rhinosinusitis (CRS), cystic fibrosis (CF), and chronic obstructive pulmonary diseases (COPD). Moreover, there is a need for novel anti-pathogen strategies to promote endogenous airway innate immunity and overcome the emergence of antibiotic-resistant pathogens [3]. One protein family that plays a central role in regulating many innate defense processes is the Akt family of kinases. We hypothesized that directly boosting Akt activity may be beneficial for several parameters of respiratory diseases. However, because Akt is also activated downstream of inflammatory receptors like toll-like receptors (TLRs) among many other kinases (described in the discussion), the specific positive or negative contributions of Akt to inflammation in airway epithelial cells has been unclear and required empirical testing.

One major target of Akt is endothelial-(e) nitric oxide synthase (NOS). Dysregulated activity of eNOS may contribute to inflammation in diseases like COPD [4] and primary ciliary dyskinesia [5]. Cytokines such as tumor necrosis factor-alpha (TNF-α) or IL-17 can inhibit eNOS through phosphorylation at T495 [6] reducing nitric oxide (NO) levels. NO is produced by the conversion of L-arginine to citrulline by NOS enzymes. Reduced eNOS function may also contribute to inflammation in endothelial and adipose cells. Activation of eNOS has anti-inflammatory effects [7] and defective activation of eNOS through loss of cystic fibrosis transmembrane conductance regulator (CFTR) as a scaffold protein may contribute to endothelial inflammation in CF [8]. Whereas high levels of NO produced by inducible-NOS (iNOS) may contribute to inflammation, lower levels of NO generated by eNOS in airway epithelial cells increase ciliary beat frequency to enhance mucociliary clearance [9]. Experimental animal models show that reduced NO in the airways of eNOS deficient mice contributes to airway hyperresponsiveness (AHR), one of the main features of asthma [10]. Inhibition of eNOS in mice increases broncho-constriction and mice deficient in eNOS are more susceptible to lung injury leading to vascular remodeling and pulmonary hypertension [11].

NO production is also antibacterial and antiviral against severe acute respiratory syndrome coronavirus (SARS-CoV-1) [12] and influenza [13]. We previously showed that acute NO production via eNOS can markedly enhance the phagocytic activity of macrophages [14], a major cell type involved in airway epithelial innate immunity. Thus, activating eNOS may be therapeutically beneficial in airway diseases both for anti-inflammatory and anti-pathogen effects. While some strategies have focused on the delivery of NO donor molecules, a less explored strategy is to pharmacologically target endogenous airway cell NO production. However, doing this requires a better understanding of mechanisms of NO generation by airway epithelial cells, which predominately express the eNOS isoform. Ca^2+^/calmodulin binding can activate eNOS. Studies have shown this to occur in airway cells downstream of GPCRs like T2R bitter taste receptors [9, 14, 15]. However, eNOS is also activated by phosphorylation at S1177 by Akt, a serine/threonine kinase that activates cell survival pathways [16]. The pleckstrin homology (PH) domain of Akt binds to phosphoinositide-3-kinase (PI3K) resulting in Akt phosphorylation at T308 and/or S473 and subsequent activation. Only a few studies have examined Akt-dependent regulation of eNOS and NO production in the airway.

Another downstream effector that may make Akt-targeting beneficial is nuclear factor erythroid 2–related factor 2 (Nrf-2) [17]. Nrf-2 is a transcription factor that resides in the cytosol and is negatively regulated by kelch-like ECH-associated protein-1 (KEAP-1). KEAP-1/Nrf-2 is a master regulator of cytoprotection against oxidative stress. Ubiquitination of KEAP-1 releases Nrf-2, which translocates to the nucleus and transcribes antioxidant genes NAD(P)H quinone dehydrogenase 1 (NQO-1) and heme oxygenase (HO-1). Nrf-2 may also have anti-inflammatory effects and protects against airway injury in mouse models [18] and Nrf-2 activators are currently in clinical trials to treat neurodegenerative diseases [19]. Although Akt can increase Nrf-2 protein levels in some tissues, it is unknown if direct Akt-dependent upregulation of Nrf-2 can reverse nuclear factor-κB (NFκB)-driven cytokine production in human airway cells. We hypothesized that direct small-molecule Akt activation can induce both NO and antioxidant Nrf-2 function, either of which may reduce inflammation in airway cells.

Defense against oxidative stress may also be particularly beneficial to preserving epithelial barrier function. The pulmonary epithelium is joined by tight junction [20] proteins creating a barrier that protects against inhaled pathogens and allows the establishment of transepithelial potential differences important for driving fluid secretion and absorption [21]. Loss of epithelial integrity is seen in lung diseases [22], including CRS [23] and CF [24]. Exposure of airway epithelial cells to oxidative insults like cigarette smoke condensate (CSC) can also cause characteristics of obstructive lung diseases and reduce barrier functions through reduction of zonula occludens 1 and 2 (ZO-1/ZO-2), major tight junction [20] proteins [25]. Further, cadmium, a toxic component of cigarette smoke and air pollutants, can increase TNF-α, a major inflammatory cytokine [26] that can disrupt epithelial integrity in the airway and contribute to inflammation.

Pharmacological activation of Akt can protect brain and renal cells from cytotoxic insults and has beneficial effects in multiple cell types [27]. While we hypothesized that Akt activation might be beneficial, this required further study since Akt might also have pro-inflammatory effects. To test if Akt activation stimulates NO production, alleviates inflammation, or protects against cadmium-induced barrier damage in airway cells, we used the small-molecule SC79 to directly activate Akt. This enables activation of Akt independent of other kinases, as would be the case during stimulation with receptor ligands such as epidermal growth factor. SC79 binds to Akt and promotes its phosphorylation by upstream kinases, and enables its cytosolic activation [27]. We measured the effects of SC79 on NO production, induction of downstream Nrf-2 targets, IL-8 production in response to TNF-α or flagellin, and barrier function during cadmium exposure.

## Materials and methods

### Reagents and solutions

Akt (#2920), phosphorylated (p)-Akt S473 (#4058), p-eNOS S1177 (#9571), Nrf-2 (#12721), actin (#3700), HRP-conjugated anti-rabbit (#7074) and anti-mouse (#7076) antibodies were from Cell Signaling Technologies (Danvers, MA, USA). Anti-eNOS (#610296) was from BD Transduction Laboratories (San Jose, CA, USA). Anti-ZO-1 (#40-2200) was from ThermoFisher (Waltham, MA, USA). L- and D-N^G^-nitroarginine methyl ester (L-NAME and D-NAME), SC79 (#14972), LY294002 (#70920), and brusatol (#30883) were purchased from Cayman Chemical (Ann Arbor, MI, USA). 4-amino-5-methylamino-2′,7′-difluorofluorescein (DAF-FM) was purchased from Life Technologies (Carlsbad, California, USA). Unless indicated below, all other reagents were from Sigma Aldrich (St. Louis, MO, USA).

### Cell-culture

A549 human type II-like (American Type Culture Collection, Manassas, VA, USA) and 16HBE14o- (SV-40 immortalized bronchial; D. Gruenert, UCSF) cells were grown in MEM containing 10% FBS and 1% penicillin/streptomycin. Primary type II alveolar cells (AEC) were obtained from ScienCell (Carlsbad, CA, USA) and were grown as described previously [28]. For Western, cells were seeded onto 6-well plates. For live cell imaging, cells were plated onto glass 8-well chamber slides (CellVis, Mountain View, CA, USA). Cells were either transfected with cerulean/cpV FRET-based sensor AktAR [29]; gift of Jin Zhang via Addgene (Watertown, MA, USA) or loaded with DAF-FM prior to stimulation with SC79. In all studies, cells were exposed to inhibitors for one hour and then stimulated with agonists as indicated. For biochemistry experiments, cells were treated with agonists and/or antagonists dissolved in serum-free media on the day of the experiment.

Culture methods for primary cells, ALI cultures, and H441 cells are in the Additional file 1.

### Live cell imaging

Imaging experiments were performed in HEPES-buffered Hank’s balanced salt solution (HBSS) in the presence of 1.8 mM Ca^2+^. To measure Akt, mTORC 1, or AMPK activity, A549 cells were transfected with AktAR, TORCAR, or ABKAR, respectively (0.5 μg DNA/well) for ~ 24 h. The next day the cells were stimulated with insulin (control) or SC79 and were imaged as described previously [9, 15] using MetaFluor software (Molecular Devices, Sunnyvale, CA, USA) and CFP/YFP filter sets (Chroma Technologies, Bellows Falls, VT) at 20x (0.8 N.A. objective) on an Olympus IX83 microscope (Olympus, Tokyo, Japan) with Hamamatsu Orca Flash 4.0 sCMOS camera.

### Immunofluorescence (IF)

Immunofluorescence was carried out as described [9, 14, 15]. A549 cells were grown in 8-well chamber slides and fixed with 4% formaldehyde at room temperature for 20 min. Cells were blocked in 5% normal donkey serum and permeabilized with 0.2% saponin. Primary antibodies (1:100) were incubated overnight at 4^ο^ C followed by secondary antibodies (Alexa 488/Alexa 546) for 2 h the next day. Images were taken on an Olympus DSU spinning disk confocal system with IX-83 microscope (Olympus, Life Sciences, Tokyo, Japan) with 60x (1.4 NA) objective using MetaMorph software (Molecular Devices, Sunnyvale, CA, USA).

### Quantitative RT-PCR

A549s were treated with TNF-α (0.1 μg/ml) or flagellin (1 µg/ml) ± SC79 (2.5 μg/ml) for 24 h then lysed with iScript RT-qPCR lysis buffer (Bio-Rad, Hercules, CA, USA); iTaq Universal SYBR Green One-Step Kit was used (Bio-Rad, Hercules, CA, USA) to measure transcripts. Primer sequences used for qPCR (5’-3’) are as follows: IL8 Forward Primer (F): TTTTGCCAAGGAGTGCTAAAGA, IL8 Reverse Primer (R): AACCCTCTGCACCCAGTTTTC, GAPDH (F): GGAGCGAGATCCCTCCAAAAT, GAPDH (R): GGCTGTTGTCATACTTCTCATGG, Nrf-2 (F): TCAGCGACGGAAAGAGTATGA, Nrf-2 (R): CCACTGGTTTCTGACTGGATGT, HO-1 (F): AAGACTGCGTTCCTGCTCAAC, HO-1 (R): AAAGCCCTACAGCAACTGTCG, NQO1 (F): GAAGAGCACTGATCGTACTGGC, and NQO1 (R): GGATACTGAAAGTTCGCAGGG. All the bar graphs represent expression relative to GAPDH. The change in CT (ΔCT) was calculated as CT_GAPDH_ – CT_target gene_, and expression relative to GAPDH was calculated as 2^−ΔCT^. Raw mean ΔCT values are reported in figure legends. Significance was tested for both 2^−ΔCT^ and ΔCT. Significance for 2^−ΔCT^ is reported in bar graphs and for ΔCT in the figure legends. Everything that was tested here and that was found to be significantly different (*p* < 0.05) was significantly different by both methods, as indicated in the figures and legends.

### Western blotting

Western blotting was carried out as described [28]. Cells were lysed with RIPA buffer containing 50 mM Tris, 150 mM NaCl, 0.1% SDS, 0.5% sodium deoxycholate, 0.1% NP-40, Phosphatase inhibitor cocktail (Sigma-Aldrich, St-Louis, MO, USA), and the cOmplete Mini Protease Inhibitor Cocktail (Roche, Indianapolis, IN, USA). After harvesting, protein concentrations were measured using Bradford assay (Bio-Rad, Hercules, CA, USA) and lysates were run on XCell-SureLock 8% mini gels (ThermoFisher Scientific, Waltham, MA, USA). After transfer to nitrocellulose, bands were visualized by Clarity Western ECL Substrate (Bio-Rad, Hercules, CA, USA). To quantify the protein levels, protein of interest and the control protein levels were analyzed using ImageJ analysis program using box density method as described previously [30]. Briefly, once the Western blot image is open in ImageJ, rectangular selections were made around each band to create a histogram. Straight-line selection tool in ImageJ was used to analyze the area for each band and was normalized to actin. Bar graphs were plotted using values from independent multiple experiments, not just the single representative blot shown.

### Nuclear fractionation

Nuclei were isolated as described [31]. Cells were lysed in 1 mL of ice-cold 0.1% NP40 in PBS using a cell scraper and collected lysates into 1.5 ml micro-centrifuge tubes. Next, the tubes were centrifuged for 10 s in a tabletop microfuge, supernatants were removed and pellets were resuspended in 900 μL of ice-cold 0.1% NP40 in PBS and mixed well with a p1000 micropipette. Afterwards, 300 μL of the lysate (whole-cell fraction) was removed and 100 μL of 4X Laemmli buffer was added to it. The remaining lysate was centrifuged for another 10 s and 300 μl of the supernatant was removed (cytosolic fraction). 100 μL of 4X Laemmli buffer was added and boiled for 1 min. After removing the remaining supernatant, the pellet was resuspended in 1 mL of ice-cold 0.1% NP40 in PBS and centrifuged for 10 s and the supernatant was discarded. The pellet was resuspended in 150 μL of 1X Laemmli buffer (Nuclear fraction) followed by Western.

### Data analysis and statistics

Statistical significance was determined in GraphPad Prism (GraphPad Software, Inc., La Jolla, CA, USA) as shown in figure legends; p < 0.05 was considered statistically significant. One-way analysis of variance (ANOVA) was used with Bonferroni posttests. All data shown in bar graphs are mean ± SEM from ≥ 3 independent experiments using cells from different passages (e.g., separate cell lysates) or lysates from different donors in the case of primary cells.

## Results

### Akt isoforms and eNOS expression in A549 and patient-derived primary nasal cells under both submerged and ALI conditions

**Fig. 1:**
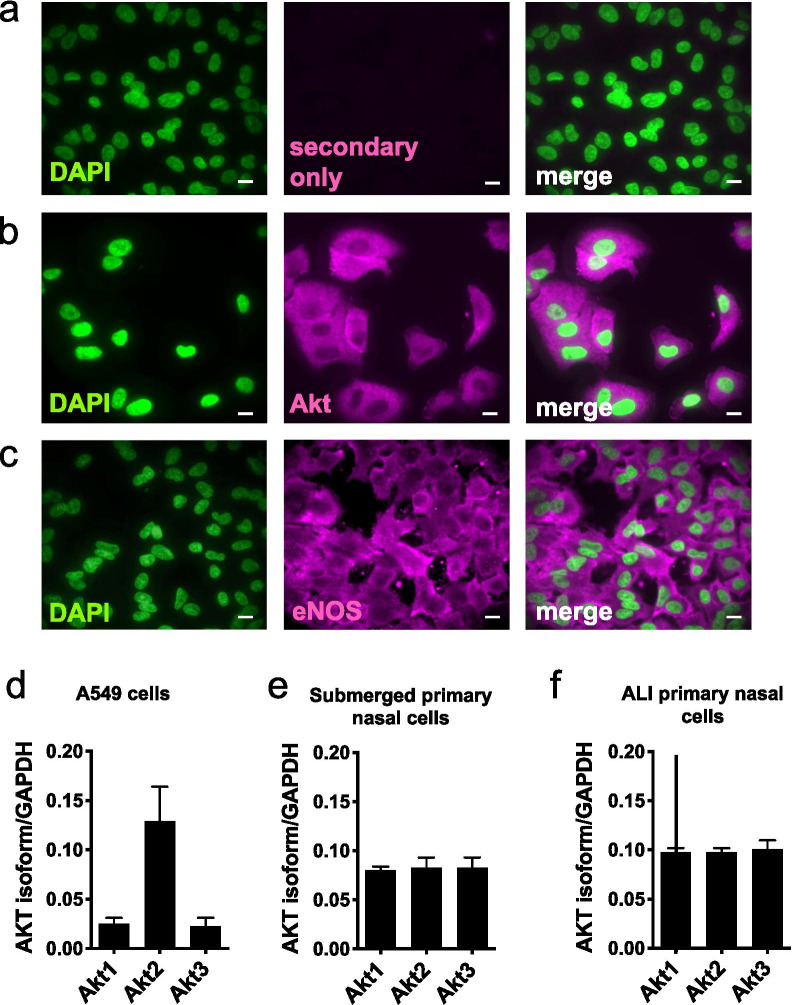
Localization and expression of Akt isoforms and eNOS in A549 and primary nasal epithelial cells. **a**–**c** IF was performed for Akt and eNOS as described. **a** Secondary antibody only control, **b** Akt, localized largely to the cytosol and **c** membrane-localized eNOS, a palmitoylated membrane-associated protein. Scale bar is 10 μm. **d**–**f** A549 cells and primary nasal cells (submerged and ALI) were lysed for qPCR to measure levels of Akt isoforms. Bars are expression relative to GAPDH (2^−ΔCT^; mean ± SEM); n = 3 experiments

Akt and eNOS expression and localization was examined in A549 alveolar-like cells using immunofluorescence. We observed cytoplasmic Akt and some partially membrane-localized eNOS (Fig. 1a, c), consistent with previous research showing eNOS is localized to the plasma membrane and/or Golgi membrane via palmitoylation [32] and in contrast with our previous observations of cytosolic iNOS in A549s [9]. As a plasma membrane control, eNOS staining partially overlapped with GLUT1 (Additional file 1: Fig. S1). Cytoplasmic/perinuclear staining likely represented Golgi or endosomal expression, as previously described for eNOS [32]. Expression of Akt was confirmed by qPCR using allele-specific primers for the three mammalian isoforms. Akt-2 mRNA is more abundant in A549s compared with Akt-1 or Akt-3 (Fig. 1d). However, human primary nasal cells (both submerged and differentiated at ALI) expressed all three isoforms of Akt (Fig. 1e, f) at relatively equal levels.

### SC79 induces Akt phosphorylation and activity in A549 cells

**Fig. 2:**
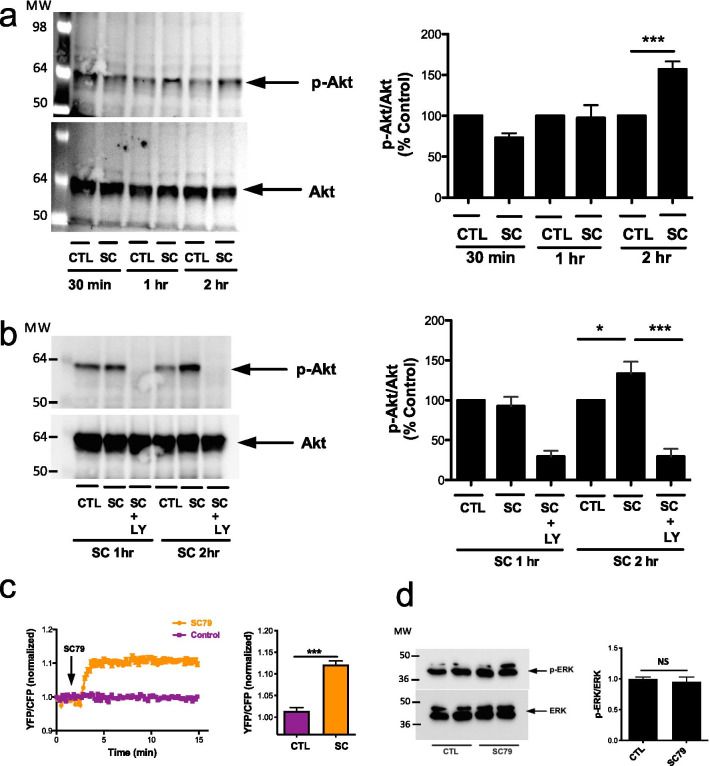
SC79 phosphorylates and activates Akt in A549 cells. **a**, **b** A549s were treated with SC79 (10 μg/ml; DMSO only as control) or PI3K inhibitor LY294002 (10 μg/ml) for 1 h before incubating with SC79 for 1 h and 2 h. Cell lysates were collected for Western. Bars are mean ± SEM; n ≥ 3 experiments. **a** ***p < 0.001 (CTL vs. SC) 2 h and **b** *p < 0.05 (CTL vs. SC) and ***p < 0.001 (SC vs SC + LY) 2 h by ANOVA and Bonferroni posttest. **c** A549s were transfected with biosensor AktAR and stimulated with SC79 (10 μg/ml). FRET (YFP/CFP emission ratio) was normalized at 14 min. Bars are mean ± SEM; n = 6. ***p < 0.001 (CTL vs. SC) by unpaired Student’s *t*-test. **d** SC79 does not induce phosphorylation of ERK1/2. A549 cells were treated with SC79 (10 μg/ml; DMSO only as control) for 2 h before harvesting for Western. Bars are mean ± SEM; n = 3 experiments

We tested whether SC79 increased phosphorylation of Akt, reflecting Akt activation, in A549s. Phosphorylated (p)-Akt (S473) was increased ~ 50% after 2 h of stimulation with SC79 (Fig. 2a). This was blocked by upstream phosphoinositide-3-kinase (PI3K) inhibitor LY294002 (IC_50_ for PI3K of ~ 1.4 µM [33]). LY294002 blocked Akt phosphorylation with SC79 at both 10 µg/ml (28 µM) (Fig. 2b) and 1 µg/ml (2.8 µM) (Additional file 1: Fig. S2). We performed live cell imaging using a sensitive FRET-based fluorescent protein biosensor of Akt activity (AktAR2), which has no cross-reactivity with other kinases such as PKA or PKC [29]. AktAR2 confirmed that SC79 upregulated Akt activity within minutes (Fig. 2c). While Akt activity with AktAR plateaued more quickly than the change in Akt phosphorylation observed via Western, this likely reflected saturation of phosphorylation sites on AktAR2.

Notably, while Akt-activating agonists like IGF-1 and TNFα increased Akt activity measured via AktAR2, the activation kinetics with IGF-1 were much slower while activation by TNFα was transient (~ 20–30 min; Additional file 1: Fig. S3a–f). SC79 induced rapid, sustained Akt activation over ≥ 18 h (Additional file 1: Fig. S3a–f). The increase in AktAR2 fluorescence with SC79 was inhibited by two Akt inhibitors, GSK690693, and MK2206, confirming that AktAR2 FRET changes reflect Akt activity (Additional file 1: Fig. S3g, h). We did not observe SC79-induced phosphorylation of ERK1/2, suggesting SC79 is specific for Akt (Fig. 2d). Confirming specificity another FRET-based indicator for AMP-activated protein kinase (AMPK), ABKAR, did not reveal any activation of AMPK (Additional file 1: Fig. S3i). Further confirming Akt activation, we saw an increase in downstream mTORC1 activity using a FRET indicator TORCAR (Additional file 1: Fig. S4). We further examined cell viability of SC79 by performing an XTT assay. As shown in Additional File 1: Fig. S5, SC79 did not reduce cellular metabolism after 24 h stimulation as measured by XTT assay (an indirect measurement of NAD(P)H production), consistent with what other research groups have shown [34].

### Small-molecule Akt activation leads to eNOS phosphorylation and production of NO

**Fig.3:**
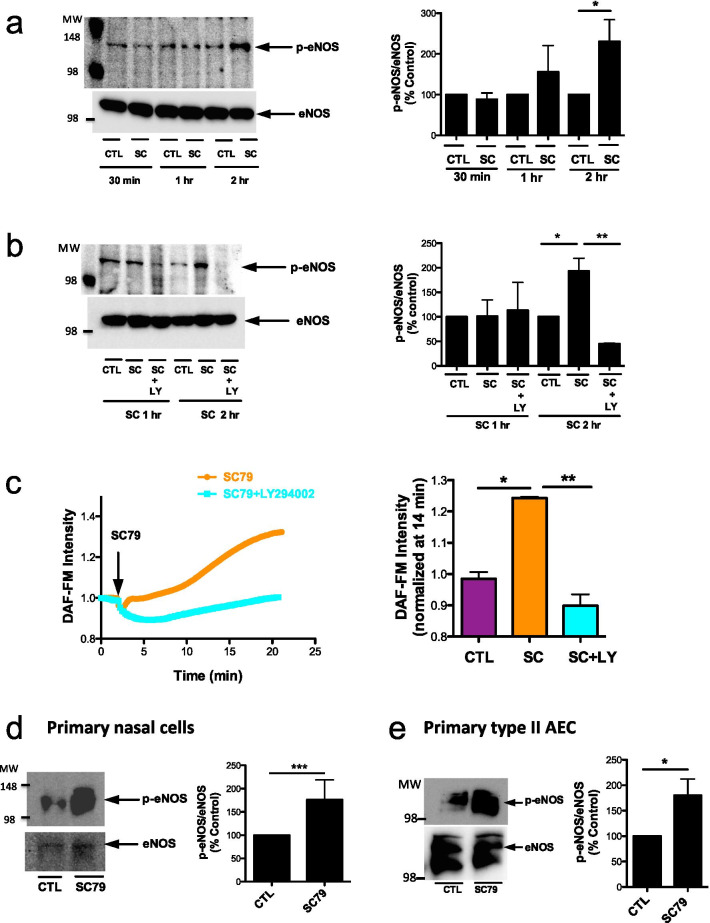
SC79 increases NO via PI3K-dependent eNOS phosphorylation in A549, primary nasal, and type II AECs. **a**, **b** Cells were treated with SC79 (10 μg/ml; DMSO only as control) for 30 min, 1 h, and, 2 h alone or with LY294002 (10 μg/ml) for 1 h before incubating with SC79. Bars are means ± SEM; n ≥ 3 experiments. **a** *p < 0.05 (CTL vs. SC) 2 h and **b** *p < 0.05 (CTL vs. SC) and **p < 0.01 (SC vs. SC + LY) 2 h by ANOVA and Bonferroni posttest. **c** Cells were loaded with 5 μM DAF-FM for 60 min and stimulated with SC79 (10 μg/ml) ± LY294002 (10 μg/ml). DAF-FM intensities were normalized 14 min after SC79. Bars are mean ± SEM; n = 3 experiments. *p < 0.05 (CTL vs. SC) and **p < 0.01 (SC vs. SC + LY) by ANOVA and Bonferroni posttest. **d** Primary nasal cells were cultured and treated with SC79 (10 μg/ml) for 2 h before lysing for Western blotting. Bars are mean ± SEM; n = 3. ***p < 0.0001 (CTL vs. SC) by unpaired Student’s *t*-test. **e** Primary type II AECs were treated with SC79 (10 μg/ml) for 2 h before lysing for Western. Bars are mean ± SEM; n = 3. *p < 0.05 (CTL vs. SC) by unpaired Student’s *t*-test

We next investigated if SC79 could induce Akt-dependent phosphorylation of eNOS at the activating site S1177 in A549s. S1177 phosphorylation of eNOS increased ~ 150% after 2 h of stimulation with SC79 by Western (Fig. 3a). This was PI3K-dependent, as it was blocked by LY294002 (Fig. 3b). Furthermore, live cell imaging experiments revealed that SC79 led to an increase in NO production measured by the fluorescent indicator DAF-FM [9, 15] (Fig. 3c). This was also blocked by LY294002, demonstrating the activation of eNOS occurs downstream of PI3K-Akt signaling. DAF-FM fluorescence increases reflected NOS activation, as they were blocked by NOS inhibitor L-NAME or NO scavenger cPTIO as well as Akt inhibition (Additional file 1: Fig. S6a–c). No evidence of reactive oxygen species production was observed (Additional file 1: Fig. S6d).

To test if Akt activation could induce NO production via eNOS in non-alveolar cells, a distal lung epithelial cell-line (H441) and dedicated innate immune cells (primary human macrophages) were treated with SC79 in the presence or absence of NOS inhibitor, L-NAME. NO production was increased with SC79 in both H441s and macrophages. Further, NO levels were blocked by L-NAME, but not with the inactive D-NAME (Additional file 1: Fig. S7, S8), confirming DAF-FM results reflect NO production by NOS, likely eNOS. Finally, we found that SC79 also induces S1177 eNOS phosphorylation in patient-derived acutely isolated primary nasal cells (Fig. 3d) and primary human type II AECs (Fig. 3e), consistent with data in A549s.

### Activation of Akt by SC79 upregulates Nrf-2 protein and expression of downstream Nrf-2 target antioxidant genes

**Fig. 4:**
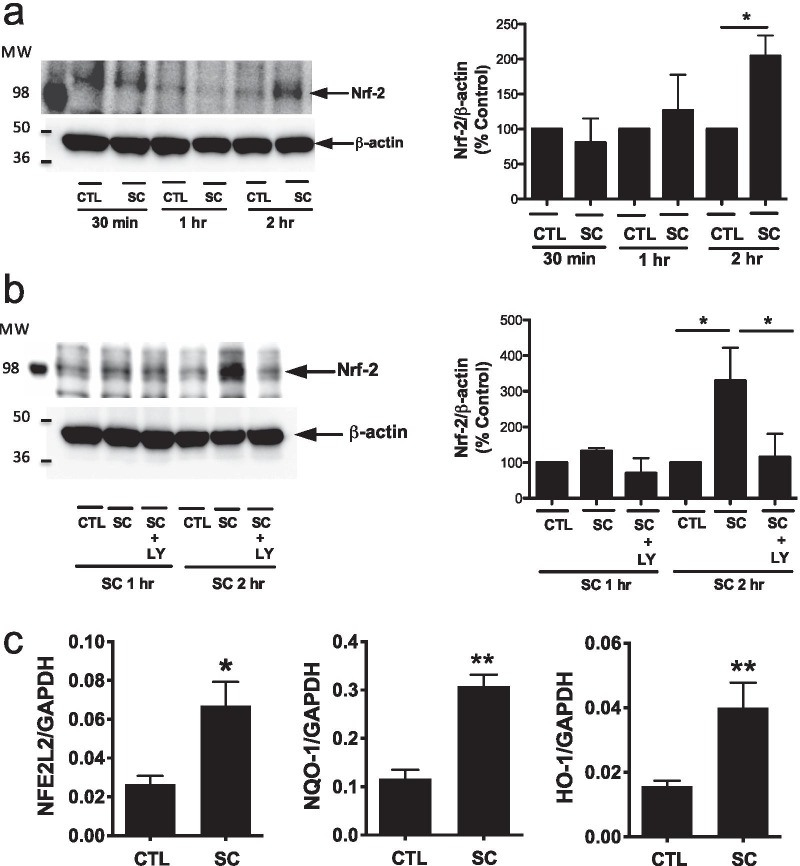
SC79 upregulates total Nrf-2 protein and downstream targets HO-1 and NQO-1. **a**, **b** A549s were treated as in Fig. 3, followed by Western for Nrf-2. Bars are means ± SEM; n ≥ 3 experiments. **a** *p < 0.05 (CTL vs. SC) 2 h and **b** *p < 0.05 (CTL vs. SC vs. SC + LY) 2 h by ANOVA and Bonferroni posttest. **c** A549s were grown and treated with SC79 (10 μg/ml) for 24 h before lysing for qPCR to measure Nrf-2 (NFE2L2), HO-1, and NQO-1. Bars show expression relative to GAPDH (2^−ΔCT^; mean ± SEM; n = 4 experiments. *p < 0.05 (NFE2L2), **p < 0.01 (HO-1) and ***p < 0.001 (NQO-1) by unpaired *t*-test. Raw ΔCT values (control vs SC) were 5.48 ± 0.26 vs 4.32 ± 0.40 (NFE2L2; *p* < 0.05 by unpaired *t*-test), 3.35 ± 0.28 vs 1.75 ± 0.12 (NQO-1; *p* < 0.001 by unpaired *t*-test) 6.09 ± 0.15 vs 4.87 ± 0.23 (HO-1; *p* < 0.01 by unpaired *t*-test)

Dysfunctional Nrf-2 may contribute to hyperinflammation observed in CF airway epithelial cells [17]. We hypothesized that direct Akt activation may increase Nrf-2 protein in airway epithelial cells. As described above, KEAP-1 binds to Nrf-2 and promotes Nrf-2 degradation by the ubiquitin–proteasome pathway. Blockade of this interaction by Akt phosphorylation reduces Nrf-2 degradation and allows Nrf-2 to translocate to the nucleus. SC79 treatment increased total Nrf-2 protein in A549 cells after 2 h (Fig. 4a), and this was dependent on PI3K (Fig. 4b), suggesting it was downstream of Akt. More importantly, treatment with SC79 also upregulated expression of the Nrf-2 (NFE2L2) gene itself as well as downstream targets HO-1 and NQO-1 at 24 h (Fig. 4c). This confirms that the increase in Nrf-2 protein and downstream targets correlate with increased Nrf-2 activity.

### SC79 Akt activation reverses TNF-α and flagellin-induced IL-8 in A549 cells and primary airway cells, likely by increasing nuclear Nrf-2

**Fig. 5:**
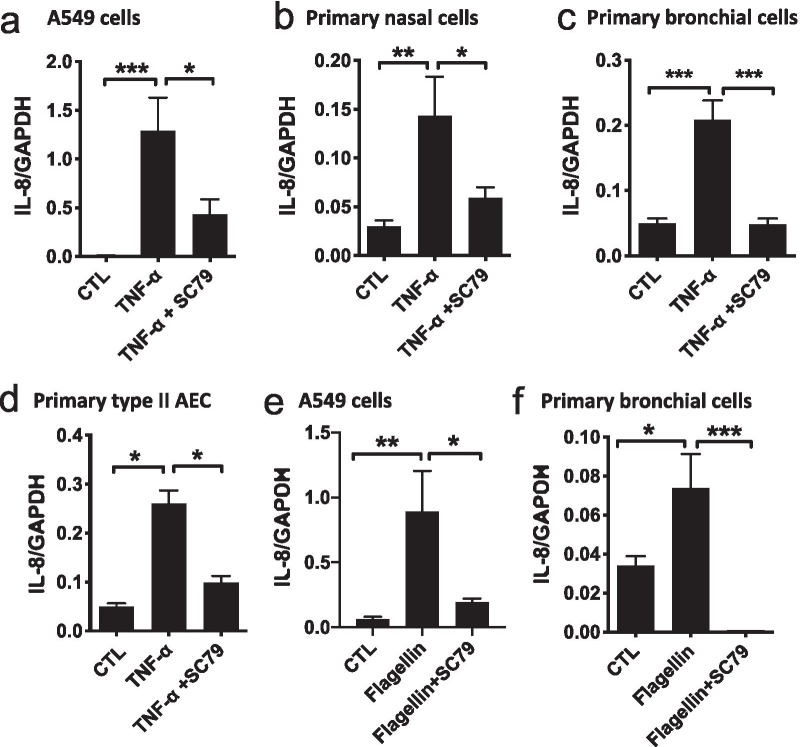
Reduction of IL-8 by SC79 in A549 and primary airway cells in response to TNF-α or flagellin. **a**–**d** Cells were treated with TNF-α (0.1 μg/ml) ± SC79 (2.5 μg/ml) for 24 h before adding RNA lysis buffer followed by qPCR. Bars are expression relative to GAPDH (2^−ΔCT^; mean ± SEM; n = 3). **a** ***p < 0.001 (CTL vs. TNF-α) and *p < 0.05 (TNF-α vs. TNF-α + SC). Raw ΔCT values were 6.99 ± 0.36 (control), 0.09 ± 0.3 (TNF-α; *p* < 0.01 vs control), and 1.95 ± 0.6 (TNF-α + SC79; *p* < 0.05 vs TNF-α alone). **b** **p < 0.01 (CTL vs. TNF-α) and *p < 0.05 (TNF-α vs. TNF-α + SC). Raw ΔCT values were 5.32 ± 0.25 (control), 3.24 ± 0.33 (TNF-α; *p* < 0.01 vs control), and 4.43 ± 0.32 (TNF-α + SC79; *p* < 0.05 vs TNF-α alone). **c** ***p < 0.001 (CTL vs. TNF-α) and ***p < 0.001 (TNF-α vs. TNF-α + SC). Raw ΔCT values were 4.47 ± 0.19 (control), 2.40 ± 0.18 (TNF-α; *p* < 0.01 vs control), and 4.70 ± 0.30 (TNF-α + SC79; *p* < 0.05 vs TNF-α alone). **d** **p < 0.01 (CTL vs. TNF-α) and *p < 0.05 (TNF-α vs. TNF-α + SC). Raw ΔCT values were 4.43 ± 0.17 (control), 2.04 ± 0.17 (TNF-α; *p* < 0.01 vs control), and 3.49 ± 0.21 (TNF-α + SC79; *p* < 0.05 vs TNF-α alone). All significance by one way ANOVA and Bonferroni posttest. **e**, **f** A549s and primary bronchial cells were treated with flagellin (1 μg/ml) ± SC79 (2.5 μg/ml) for 24 h and qPCR was performed. Bars are mean ± SEM; n = 3. **e** **p < 0.01 (CTL vs. flagellin) and *p < 0.05 (flagellin vs. flagellin + SC). Raw ΔCT values were 4.27 ± 0.26 (control), 0.72 ± 0.37 (flagellin; *p* < 0.01 vs control), and 2.53 ± 0.23 (flagellin + SC79; *p* < 0.05 vs flagellin only). **f** *p < 0.05 (CTL vs. flagellin) and ***p < 0.001 (flagellin vs. flagellin + SC). Raw ΔCT values were 5.07 ± 0.24 (control), 4.21 ± 0.34 (flagellin; *p* < 0.05 vs control), and 14 ± 0.57 (flagellin + SC79; *p* < 0.001 vs flagellin only). All significance by one-way ANOVA and Bonferroni posttest

TNF-α is an important pro-inflammatory cytokine in the airway [26]. While the TNF-α receptor activates Akt, this activation is lesser in magnitude than SC79 and is transient (Additional file 1; Fig. S3c). Treatment of A549s, primary nasal or bronchial epithelial cells, or primary type II AECs with TNF-α for 24 h induced transcription of IL-8 (Fig. 5a–d), a chemokine/cytokine regulated by NF-κB that is elevated in inflammatory airway diseases. Akt activation by SC79 reduced TNF-α-induced IL-8 by > 50%. We also stimulated A549 and primary bronchial cells with flagellin, a bacterial product that induces NF-κB/IL-8 via activation of TLR-5 [35]. Similar to TNF-α, flagellin-induced IL-8 levels were almost completely reduced by SC79 (Fig. 5e, f).

**Fig. 6:**
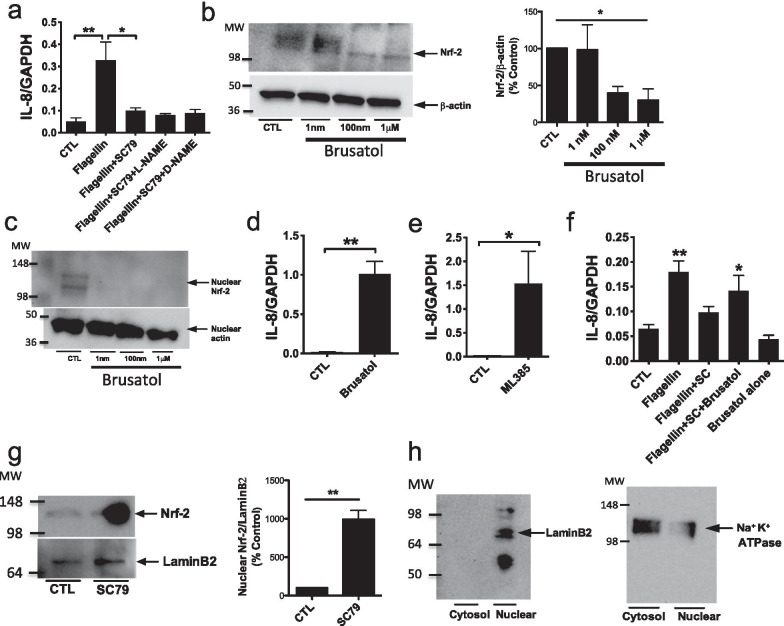
The Akt-activated Nrf-2 likely reverses the SC79 inhibition of IL-8 transcription in A549 cells. **a** A549s were treated with flagellin (1 μg/ml) ± SC79 (2.5 μg/ml) ± L-NAME (10 μM) or D-NAME (10 μM) for 24 h and qPCR was performed. Bars are expression relative to GAPDH (2^−ΔCT^; means ± SEM; n = 4 experiments).**p < 0.01 (CTL vs. flagellin) and *p < 0.05 (flagellin vs. flagellin + SC) by ANOVA and Bonferroni posttest. Raw ΔCT values were 4.79 ± 0.30 (control), 2.02 ± 0.33 (flagellin; **p < 0.01 vs control), 3.46 ± 0.17 (flagellin + SC79; *p < 0.05 vs flagellin only), 3.71 ± 0.12 (flagellin + SC79 + L-NAME), 3.69 ± 0.22 (flagellin + SC79 + D-NAME). **b** Brusatol (≥ 100 nM) degrades total Nrf-2 in A549 cells, and this is sufficient to increase IL-8 levels. Cells were grown and treated with brusatol at 1 nm, 100 nm, and 1 μM for 2 h before lysing the cells for Western. Bars are means ± SEM; n = 3 experiments; *p < 0.05 (CTL vs. 1 μM) by ANOVA and Bonferroni posttest. **c** Brusatol reduces nuclear Nrf-2 at ≥ 1 nM. A549 cells were grown and were treated with brusatol at 1 nm, 100 nm, and 1 μM for 2 h. Cell lysates were collected and centrifuged to separate nuclear fractions as described in the text. Actin shown as a loading control. Representative of 3 experiments showing complete loss of nuclear Nrf-2. **d** Nrf-2 inhibition alone with brusatol at higher concentrations increases IL-8 in A549 cells. A549 cells were grown and treated with 1 μM brusatol for 24 h. Cells were lysed for qPCR. Bars are expression relative to GAPDH (2^−ΔCT^; means ± SEM; n = 3 experiments). *p < 0.001 (CTL vs. 1 μM brusatol) by unpaired *t*-test. Raw ΔCT values (control vs brusatol) were 5.96 ± 0.19 vs 0.16 ± 0.21 (*p* < 0.001 by unpaired *t* test). **e** ML385 alone (1 μM)) also increases IL-8 in A549 cells. Bars are expression relative to GAPDH (2^−ΔCT^; means ± SEM; n = 3 experiments). *p* < 0.001 (CTL vs. 1 μM) by unpaired *t*-test. Raw ΔCT values (control vs ML385) were 5.88 ± 0.23 vs 0.26 ± 0.78 (*p* < 0.001 by unpaired *t* test) **f** Brusatol (1 nM) partially inhibits SC79 reduced IL-8 in A549 cells. A549 cells were grown and treated with flagellin (1 μg/ml) ± SC79 (2.5 μg/ml), and ± brusatol (1 nM) for 24 h and qPCR was performed. Bars are expression relative to GAPDH (2^−ΔCT^; means ± SEM; n = 4 experiments). Significance determined by one-way ANOVA with Dunnett’s posttest; * *p* < 0.05 and **p < 0.01 vs control. Raw ΔCT values were 4.06 ± 0.15 (control), 2.71 ± 0.19 (flagellin; *p* < 0.01 vs control), 4.16 ± 0.36 (flagellin + SC79), 3.26 ± 0.29 (flagellin + SC79 + brusatol; *p* < 0.01 vs control), 5.55 ± 0.46 (brusatol alone). **g** Nrf-2 is upregulated in the nuclear fractions in response to SC79. A549 cells were treated with SC79 (10 μg/ml; DMSO only as control) for 2 h. Cell lysates were collected and centrifuged to separate nuclear fractions. Bars are means ± SEM; n = 3 experiments. **p < 0.01 (CTL vs. SC) by unpaired Student’s *t*-test. **h** Western of LaminB2 for nuclear fractions and plasma membrane Na^+^K^+^-ATPase for cytosolic fractions

To investigate the mechanism(s) by which SC79 reduces IL-8 transcription in response to flagellin, we first tested a pharmacological inhibitor of eNOS, L-NAME. Inhibition of eNOS did not reverse the SC79-induced reduction of IL-8 (Fig. 6a), suggesting eNOS and NO production was not critical. However, Nrf-2 inhibitor brusatol, which causes degradation of Nrf-2 at 100 nM–1 µM (Fig. 6b), increased IL-8 transcription (Fig. 6c). This is consistent with other studies showing Nrf-2 reduces inflammation in animal models [17]. A concentration of brusatol below the threshold able to degrade Nrf-2 (1 nM; consistent with other studies [36]) was not sufficient to increase IL-8 levels [37] (Fig. 6c). Another Nrf-2 inhibitor, ML385, was also sufficient to increase IL-8 levels (Fig. 6c), supporting that reducing Nrf-2 activity is sufficient to increase IL-8 transcription in airway cells. Therefore, we hypothesize that Nrf-2 activity reduces IL-8 secretion, as shown in gastric cells stimulated with α-lipoic acid [38].

To determine if SC79 stimulation increases nuclear Nrf-2, we biochemically separated nuclear and non-nuclear fractions after treating A549 cells with SC79. Nrf-2 protein levels were significantly elevated in the nucleus with SC79 treatment (Fig. 6d). LaminB and Na^+^ATPase were used as controls for the nuclear and the non-nuclear/cytosol fractions, respectively (Fig. 6e). Using both immunofluorescence of endogenous Nrf-2 (Additional file 1: Fig. S9) and fluorescence of GFP-tagged Nrf-2 (Additional file 1: Fig. S10), we observed increases in nuclear Nrf-2 over 2 h stimulation with SC79. After 18 h, nuclear Nrf-2 was observed in cells stimulated with SC79 (Additional file 1: Fig. S9 and S10). TNF-α and flagellin did not induce visible nuclear Nrf-2 levels, but nuclear Nrf-2 was increased with TNF-α or flagellin ± SC79 (Additional file 1: Fig. S9 and S10). Together with prior studies showing the inhibition of IL-8 transcription by Nrf-2 in other cell types (e.g., [38]), we hypothesize that nuclear Nrf-2 elevation downstream of SC79-induced Akt activation is responsible for the reduced IL-8 during TNF-α or flagellin stimulation.

### SC79 Akt activation also protects airway cells against cadmium-induced airway barrier dysfunction

We also investigated if SC79 could reverse the loss of epithelial barrier function induced by oxidative stressors. To model epithelial injury during oxidative stress, we used cadmium, a highly reactive component of cigarette smoke and air pollutants that activates oxidative stress and can disrupt airway tight junctions in vitro [39]*.* Exposure of bronchial ALI cultures to cadmium caused a profound reduction in transepithelial electrical resistance (TEER) which was reversed with Akt activation (Additional file 1: Fig. S11a). Cadmium significantly reduced the TJ protein ZO-1, which was similarly reversed by SC79 (Additional file 1: Fig. S11b). To further investigate the role of Akt in regulating ZO-1, we incubated ALI cultures with a PI3K inhibitor in addition to SC79. Inhibiting PI3K caused a significant decrease in ZO-1 (Additional file 1: Fig. S11c), suggesting Akt is critical for maintaining epithelial barrier function, even in the absence of oxidative stress.

## Discussion

Growing evidence suggests sustained activation of PI3K/Akt may be protective in various disease models [27, 40]. In a rat model of brain injury, direct Akt activation is neuroprotective via anti-apoptotic and antioxidant proteins [41]. Further, oxidative stress-related cell death was reversed by activation of Akt in human neurons [42]. Moreover, in primary mouse hepatocytes, LPS induced TNF-α mediated liver injury was reversed by SC79 in a PI3K-dependent manner [43].

However, the specific contributions of Akt to airway epithelial inflammation have been unclear. Few studies have investigated the non-cancerous functions of Akt signaling in human airway cells, including if direct Akt activation may be beneficial for parameters of inflammatory respiratory diseases. Akt is largely understudied in the airway compared with other signaling pathways such as calcium, PKC, and cAMP/PKA. To examine functions of Akt in airway cells, we used SC79, a small molecule originally identified in cell-based high throughput screening to activate all three Akt isoforms in multiple non-lung cell types [27]. We found that SC79 increased phosphorylation of Akt at S473 in airway cells, often used as a correlate for activation. We observed faster onset of Akt activity via a protein biosensor, possibly caused by earlier phosphorylation of Akt at other activating sites (e.g., T308) or phosphorylation-independent activity stimulated by SC79. However, many effects observed here are blocked by LY294002, suggesting that either (1) Akt phosphorylation by PI3K is important for SC79 effects reported here or (2) the interaction with PI3K localizes Akt at the appropriate signaling domain. Future work will examine the localization of Akt in airway cells and how this affects anti-inflammatory signaling and/or NO production.

**Fig. 7:**
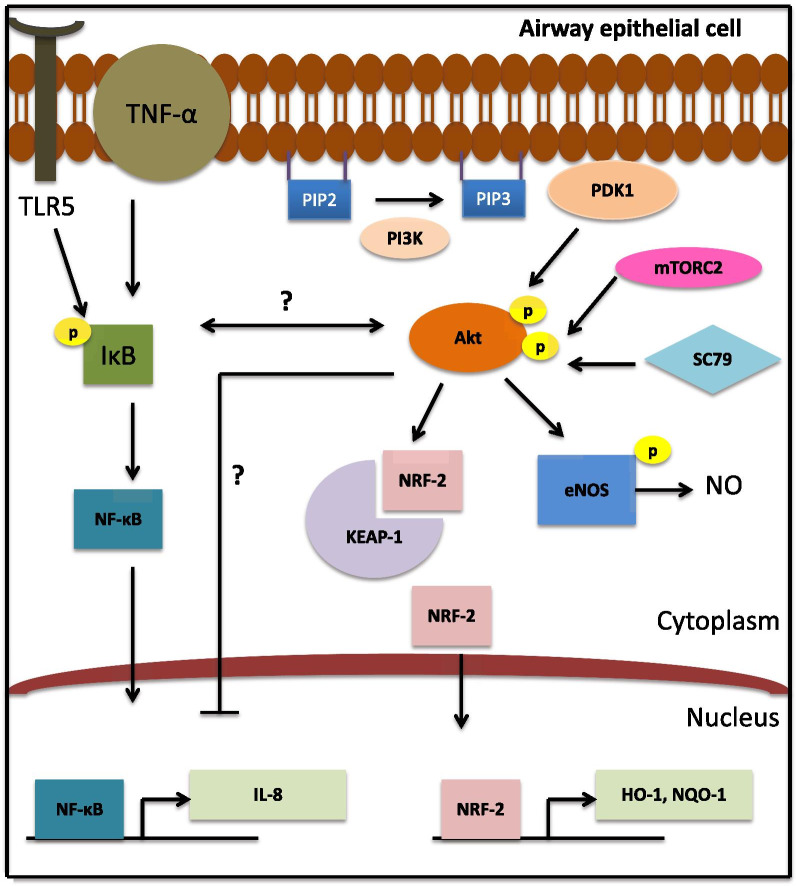
Potential effects of SC79-activated Akt signaling in airway cells. Akt activation by SC79 upregulates eNOS phosphorylation to activate the production of NO. It also upregulates Nrf-2 and downstream antioxidant genes. Both TNF-α and flagellin-induced IL-8 levels were lowered by activating the Akt pathway, likely through Nrf-2, affecting NF-κB regulated IL-8 transcription

We demonstrated that SC79 activation of Akt has two downstream targets important for innate defense in airway epithelial cells: phosphorylation of eNOS and upregulation of Nrf-2 (Fig. 7), which likely have beneficial effects against inflammation and oxidative stress. Akt activation downstream of PI3K causes eNOS phosphorylation at S1177 and upregulates NO production in A549, primary nasal epithelial cells, primary human type II AECs, H441 distal lung epithelial cells, and primary human macrophages. This Akt-increased eNOS-mediated NO production may have beneficial effects in various types of lung diseases. Loss of eNOS function has been proposed as both the cause and result of airway inflammation. Lungs of patients with pulmonary hypertension showed decreased levels of eNOS compared with control lungs [44], and eNOS may be dysregulated in COPD, leading to increased inflammation [4]. Boosting NO production by Akt activation may be useful in these diseases.

NO is also important for innate immunity. In an earlier publication from our laboratory, we demonstrated that eNOS production of NO enhances phagocytosis in human macrophages [14]. Similarly, in ciliated cells, eNOS-produced NO induces ciliary beating, facilitates mucus clearance, has bactericidal, and likely anti-viral properties [45]. Activating Akt may induce multiple beneficial effects through NO. Notably, suppression of Akt has been tied to apoptosis in airway infection by viruses such as SARS-CoV-1 [46]. Data here suggest this hypothesis however warrants further testing in in vitro and in vivo infection models. Our current study contributes to understanding of the signaling pathways regulating eNOS, relevant to human airway inflammation and infection.

We found that SC79-activated PI3K/Akt signaling increases Nrf-2 protein and transcription of downstream antioxidant genes HO-1 and NQO-1. Downstream target genes of Nrf-2 protect against oxidative stress caused by cigarette smoke in lung cells, and activators of Nrf-2 were suggested to be beneficial in COPD [47]. Lungs from COPD patients had lower levels of Nrf-2 regulated genes and higher levels of oxidative stress markers compared with normal lungs [48]. Interestingly, knockdown of Nrf-2 in human nasal cells significantly increased influenza A entry and replication, while Nrf-2 activators reduced replication [49]. Activation of Nrf-2 reduced allergic inflammation in a mouse model, further supporting the protective effects of Nrf-2. Moreover, inhibition of the PI3K/Akt pathway suppressed Nrf-2 targeted genes in bronchoalveolar lavage fluid in hypoxic mice [18]. Activation of Akt by SC79 has shown to protect oxygen and glucose-deprived myocardiocytes through reduction of oxidative stress by Nrf-2 [50]. Together with previous studies, our study suggests there may be an antioxidant and anti-inflammatory benefit to boosting Nrf-2 via small-molecule Akt activation in airway diseases.

We found that SC79 reduces IL-8 production during exposure to TNF-α or flagellin, both of which signal through the master inflammatory regulator NF-κB. In gastric epithelial cells, bacteria-induced IL-8 and high levels of ROS were reduced by HO-1 via Nrf-2 [38]. Our data similarly suggest that decreased IL-8 levels by SC79 can be reversed by blockade of Nrf-2 by brusatol. However, brusatol results must be interpreted with caution, as inhibition of the Nrf-2 pathway alone by brusatol or ML385 at higher concentrations elevated IL-8 transcript. Despite the fact that we used a lower concentration that did not have effects alone to partially reverse the SC79 effect, this limits the usefulness of these data. Future studies with Nrf-2 knockout/knockdown are necessary to further clarify the importance of Nrf-2 in the effects of SC79 observed here, given the caveats of the pharmacological inhibitors. The brusatol experiments are suggestive that Nrf-2 is required, but not definitive.

Pharmacological studies have suggested anti-inflammatory cross-talk between Nrf-2 and NF-κB pathways, although the exact dynamics of this cross-talk is yet to be elucidated [17]. In endothelial cells, induction of HO-1 inhibited NF-κB-mediated transcription of adhesion molecules such as vascular cell adhesion molecule-1 and E-Selectin [51]. It is likely that SC79-induced Nrf-2 reduces NF-κB-mediated transcription of IL-8, though this requires further experimental confirmation. Notably, we found that SC79 diminishes IL-8 levels across a spectrum of primary nasal, bronchial, and type II AECs. While some studies have shown PI3K activation and Akt phosphorylation downstream of inflammatory TLR activation, this is typically transient (< 30 min–1 h) [52]. In contrast, more prolonged activation of Akt during GPCR stimulation has been shown to reduce TLR4/LPS signaling in monocytes and macrophages [53]. We hypothesize that the prolonged (> 24 h) Akt activation underlies the anti-inflammatory effects of SC79 here.

Boosting antioxidant genes via Nrf-2 by targeting Akt may be beneficial to alleviate oxidative stress in airway diseases [54]. We modeled oxidative stress using cadmium, a major carcinogenic component of cigarette smoke with a half-life of > 25 years in the human body [55]. Cadmium is an air pollutant but also present in foods, such as shellfish and mushrooms. After cadmium exposure, about 5% is absorbed from the digestive tract, whereas lung absorption is much higher, approximately 90% of the inhaled dose [56]. Exposure to cadmium disrupts tight junctions via oxidative stress [39]. We found cadmium-reduced TEER and tight junction protein ZO-1 levels were significantly ameliorated by SC79. Moreover, baseline ZO-1 levels were regulated by Akt/PI3K, supporting the importance of Akt in barrier integrity.

## Conclusions

In summary, we have demonstrated that direct small-molecule Akt activation may exert anti-inflammatory effects in airway cells through upregulation of Nrf-2. We further showed PI3K/Akt-mediated upregulation of eNOS/NO that may have anti-pathogenic effects. Activation of PI3K/Akt signaling might also enhance epithelial barrier function by increasing ZO-1 levels. Hence, direct Akt activation by compounds such as SC79 might protect against injury induced by inflammatory and/or obstructive airway disease modifiers such as cigarette smoke, air pollutants, or pathogen exposure. We also hypothesize that targeting upstream Akt rather than downstream Nrf-2 may result in greater benefit from anti-bacterial (eNOS activation) and/or barrier-enhancing (ZO-1 increasing) effects reported here. We hypothesize that direct activation of Akt through a potent small molecule like SC79, possibly delivered to the airway through a nebulizer or nasal spray, may have protective pro-survival, antioxidant, and/or anti-inflammatory effects against viral infection or bacteria susceptible to NO (e.g., *Pseudomonas* infections in CF).

## Supplementary Information

**Additional file 1: Figure S1.** Co-localization and expression of glut1 and eNOS in A549 cells. IF was performed for GLUT1 (SLC2A1) and eNOS as described in the main text. (a) Secondary antibody only control, (b) glut-1 (cyan) and eNOs (magenta) localized largely to the plasma membrane, and (c) Line-scan showing colocalization of glut1 and eNOS. Scale bar is 10 μm. **Figure S2.** Inhibition of PI3K with 1μg/ml LY294002 reverses SC79-induced p-Akt. A549s were treated with SC79 (10μg/ml; DMSO only as control) or PI3K inhibitor LY294002 (1μg/ml) for 1 h before incubating with SC79 for 2 h. Cell lysates were collected for Western. Bars are means ± SEM; n = 3 experiments. (a) *p<0.05 (CTL vs. SC) and **p<0.01 (SC vs. SC+LY) by ANOVA and Bonferroni posttest. **Figure S3.** SC79-induced Akt activity, visualized by AktAR, is sustained over 18 hours. (a) AktAR is constructed from cyan fluorescent protein (CFP) variant cerulean and YFP-variant circularly permutated (cp)Venus with an E172 mutation surrounding a forkhead-associated domain (FHA1) used as the phosphorylated amino acid binding domain and the sequence surrounding Thr-24 of FOXO1 as an Akt substrate (10). (b) Phosphorylation by Akt causes a conformational shift that increases FRET between the CFP and YFP moieties that increases YFP emission and decreases CFP emission with CFP excitation. (c) Representative trace showing AktAR fluorescence changes. After transfection with AktAR (Lipofectamine 3000, 24 hrs., as described in the text), cells on chambered coverglass were imaged on an inverted microscope (Olympus, Tokyo Japan; 20x 0.8 NA objective) with motorized programmable stage (Prior Scientific, Rockland MA) and standard CFP/YFP emission filters in motorized filter wheels (Lambda LS, Sutter Instruments, Novato California). CFP and YFP emission (both with CFP excitation) was collected every 2 min for 3 hours. Cells were stimulated with 1 μg/ml SC-79, 50 ng/ml IGF-1, 0.1 μg/ml TNFa, or buffer alone after 8 min. After overnight incubation on the stage of the microscope, cells were also imaged at 17.5-18 hours (note break in axis). Traces suggest sustained Akt activity with SC-79 or IGF-1 but not TNFa or control (media only). (d) Bar graph of n = 4 experiments as in c. Data points show AktAR YFP/CFP em from individual experiments at 3 time points: 0.5, 2, and 18 hrs. Significance by one-way ANOVA with Dunnett’s posttest comparing each stimulated conditions (SC79, IGF1, or TNFa) to control unstimulated condition at each time point. Bars are mean ± SEM; *p<0.01. (e) Trace showing increase in AktAR YFP/CFP emission with 10 nM IGF-1 but not 10 nM insulin. IGF-1 YFP/CFP emission increase was also blocked by 1 μg/ml LY294002 (PI3K inhibitor). This fits previous studies showing IGF-1 is a more potent activator of Akt signaling than insulin in A549s (11). (f) Bar graph showing independent experiments (n = 3) and mean ± SEM from experiments as in e. YFP/CFP emission at 2 hours is shown. Significance by one-way ANOVA with Dunnett’s posttest comparing all values to IGF-1. (g) Representative traces showing AktAR YFP/CFP emission during SC79 stimulation with Akt inhibitors GSK690693 (1 μM) or MK2206 (5 μM). DMSO alone (0.1% was used as vehicle control. SC79 increased AktAR YFP/CFP emission, but this was reduced by Akt inhibitors. DMSO alone had no effect. (h) Bar graph showing mean ± SEM and individual values from experiments as in g. Peak AktAR YFP/CFP em is shown. Significance by one-way ANOVA comparing all values to SC79 (Dunnett’s posttest); *p<0.01. Together, these data support that SC79 induces sustained Akt activation in A549s. (i-j) Representative trace (i) and bar graph showing change in ABKAR, a FRET reporter of AMP-activated protein kinase (AMPK) activity. AMPK is negatively regulated by Akt phosphorylation that prevents the accessibility of LKB1 or CAMMK to the activating T172 AMPK phosphorylation site (12-15) we see a reduction in YFP/CFP emission (reflecting AMPK activity) with SC79 but an increase in YFP/CFP emission (reflecting increased AMPK activity) with ionomycin, which was used in the original ABKAR paper (16) to activate AMPK by CaMKKβ (17). Significance in j by Student’s t test; **p<0.01. **Figure S4.** SC79 activates mTORC1 via Akt in A549 cells. (a) Activation of Akt results in phosphorylation of TSC2 at S939, S981, and S1462 recruit 14-3-3 to TSC2 and disrupt the TSC1/TSC2 dimer, allowing increased mTORC1 activity (18-20). In other words, Akt inhibition of TSC1/TSC2 dimerization reduces TSC1/TSC2 inhibition of mTORC1. Thus, Akt activation can enhance mTORC1 activity. (b) We visualized mTORC1 activity using fluorescent biosensor TORCAR (21, 22); Addgene plasmid #64927) transfected into A549 cells. TORCAR contains 4EBP1, a mTORC1 phosphorylation substrate, flanked by cyan fluorescent protein (CFP) variant cerulean and yellow fluorescent protein (YFP) variant YPet. (c) Phosphorylation of the 4EBP1 in TORCAR at T37 and T46 results in a conformation shift in the TORCAR protein that decreases FRET between the CFP and YFP. This increases CFP emission and reduces YFP emission during CFP excitation. (d) After transfection with TORCAR (Lipofectamine 3000, 24 hrs., as described in the text), cells on chambered coverglass were imaged on an inverted microscope (Olympus, Tokyo Japan; 20x 0.8 NA objective) with motorized programmable stage (Prior Scientific, Rockland MA) and standard CFP/YFP emission filters in motorized filter wheels (Lambda LS, Sutter Instruments, Novato California). CFP and YFP emission (both with CFP excitation) was collected every 2 min for 3 hours. After overnight incubation on the stage of the microscope, cells were also imaged at 17.5-18 hours (note break in axis). Cells were stimulated with 1 μg/ml SC-79, 50 ng/ml IGF-1, 0.1 μg/ml TNFa, or buffer alone after 8 min. Note sustained mTORC1 activity with SC-79 and IGF-1 but not TNFa. However, onset of mTORC1 activity was more rapid with SC-79 compared with IGF-1. Traces shown are from the same single experiment where 8 transfected cells were imaged per condition. Each condition was from a separate well of the same chamber slide. (e) Bar graph of n = 3 experiments as in (d). Data points show TORCAR CFP/YFP em from individual experiments at 3 time points: 0.5, 2, and 18 hrs. Significance by one-way ANOVA with Dunnett’s posttest comparing each stimulated conditions (SC79, IGF1, or TNFa) to control unstimulated condition at each time point. Bars are mean ± SEM; *p<0.01. (f) Representative experiment as in d but with SC79 ± Akt inhibitor MK2206 (5 μM), PI3K inhibitor LY294002 (1 μM), or mTOR inhibitor rapamycin (0.5 μM) over 3 hrs. Cells were pretreated with inhibitors for 20 min before the addition of 1 μg/ml SC79. (g) Bar graph showing data from 3 independent experiments as in f for TORCAR CFP/YFP emission at 2 hours. TORCAR CFP/YFP emission ratio was reduced in the presence of Akt, PI3K, or mTOR inhibitor. Significance by one way ANOVA with Dunnett’s posttest comparing all values to SC79 alone. (h) Representative experiment showing TORCAR CFP/YFP emission during stimulation with 10 nM insulin or 10 nM IGF1 ± rapamycin. Note increase in TORCAR CFP/YFP emission with IGF1 but not insulin and inhibition with rapamycin. (i) Bar graph showing independent experiments and mean ± SEM from experiments as in h. Significance by one-way ANOVA with Dunnett’s posttest comparing all values to IGF-1; **p<0.01. (j) Representative experiment showing CFP/YFP emission during SC79 stimulation in cells transfected with TORCAR or TORCAR T/A (Addgene plasmid #64928), which has mTORC1 phosphorylation sites mutated. Note increase in TORCAR but not TORCAR T/A fluorescence, showing that the changes observed depend on phosphorylation of this site, fitting with the rapamycin data above. (k) Bar graph of independent experiments as in j showing individual experiments and mean ± SEM. Significance by Student’s ttest; ** p<0.01. Together, these studies confirm SC79 induces sustained activation of Akt by showing downstream sustained activation of mTORC1 in A549s. **Figure S5.** SC79 does not reduce cell metabolism at 24 h and 48 h. A549 cells were treated with SC79 (2.5μg/ml; DMSO only as control). After 24 or 48 hours, 100μl of XTT/PMS solution was added to each well for 1 h and the absorbance was measured at a wavelength of 450 nm and background absorbance was measured at 660 nm. Background absorbance was subtracted from signal absorbance to gain normalized values. Bars are means ± SEM; n = 3. The two groups were not significantly different by Student’s t test. **Figure S6.** SC79 induced DAF-FM increases reflect Akt-dependent NO production in A549 cells. (a) Images of A549 cells before and after SC79 (1 μg/ml) stimulation. Note no overt changes in cell morphology after SC79 stimulation. DAF-FM images were taken at 20x (0.8 NA objective) using standard FITC excitation filters as described in the methods. (b) Left shows DAF-FM traces from separate individual experiments (mean ± SEM of 10-20 cells) during stimulation with SC79 in the absence of inhibitors (control) or after 45 min pretreatment with 10 μM L-NAME (nitric oxide synthase inhibitor) or D-NAME (inactive control). Note increase in DAF-FM fluorescence in D-NAME treated cells is comparable to control while increase in LNAME treated cells is significantly blunted, supporting that DAF-FM changes reflect NOS activity. DAF-FM fluorescence increases were also blocked in the presence of 10 μM cPTIO (NO scavenger, added at the beginning of the experiment), further supporting that DAF-FM changes reflect NO production. DAF-FM fluorescence increase was also reduced in the presence of PI3K inhibitors LY294002 or wortmannin as well as Akt inhibitor MK2206. Together, these data suggest that DAF-FM changes during SC79 stimulation reflect Akt-induction of NOS activity. Right shows the bar graph of data points (DAF-FM fluorescence after 15 min) from individual experiments (n = 4-6 per condition). Bars are mean ± SEM. Control is SC79 alone. Significance by one-way ANOVA with Dunnett’s posttest comparing all values to SC79 alone (control); ** p<0.01. (c) A549 cell were untransfected or transfected with empty vector (pcDNA3.0) or pcDNA containing Wt or dominant negative K179M Akt (Addgene plasmid # Plasmid #16243 or #734058; (23, 24)). Left shows traces from individual separate DAF-FM experiments (10-20 cells imaged per condition). NO production was reduced with K179M Akt expression. Right shows data points from individual experiments (n = 6 per condition). Significance by one-way ANOVA with Dunnett’s posttest comparing all values to untransfected control; **p<0.01. This further supports that increase in DAF-FM observed with SC79 are dependent on Akt signaling. (d) A549s were similarly loaded with CellRox Deep Red (ThermoFisher Scientific; general reactive oxygen species-sensitive dye) for 30 min in the presence of SC79 or H2O2 (positive control to induce oxidative stress). No change in Cell Rox Deep Red fluorescence was observed with SC79, further supporting that DAFFM changes above reflect NO and not another oxidative species. Images are from representative experiments, taken using 20x (0.8 NA) objective and standard Cy5 excitation and emission filters. Bar graph shows mean ± SEM from individual independent experiments (n = 4 each). Significance determined by one way ANOVA with Dunnett’s posttest comparing all values to control (unstimulated); **p<0.01. **Figure S7.** SC79 increased NO through eNOS in human distal lung epithelial cell-line H441. Cells were grown in 24-well chamber slides and were loaded with DAF-FM (5 μM) for 60 min before stimulating with SC79 (10 μg/ml). (a) Control, (b) D-NAME, inactive control for L-NAME (10 μM) and, (c) eNOS inhibitor L-NAME (10 μM). DAF-FM intensities were normalized at 15 min. Non-specific NO donor S-nitroso-N-acetyl-D, Lpenicillamine (SNAP, 10 μM) was added at the end as a positive control. (d) Bars are means ± SEM; n ≥ 3 experiments. **p<0.01 (Control vs. L-NAME) by ANOVA and Bonferroni posttest. **Figure S8.** Induction of NO via eNOS in human primary MFs with SC79. MFs were loaded with DAF-FM and were stimulated with SC79 (10μg/ml). (a) Control, (b) D-NAME, inactive control for L-NAME (10 μM) and, (c) eNOS inhibitor L-NAME (10 μM). DAF-FM intensities were normalized at 10 min. (d) Bars are means ± SEM; n=3 experiments. *p<0.05 (Control vs. L-NAME) by ANOVA and Bonferroni posttest. **Figure S9.** SC79 induces nuclear Nrf-2 accumulation by immunofluorescence of endogenous Nrf-2. (a) A549 cells were grown in plastic 24 well plates and incubated for 2 hours in Hank’s balanced salt solution (HBSS) with 20 mM HEPES in the presence of vehicle only (0.1% DMSO; control), 1 μg/ml SC-79, or 1 μM SC79 + 5 μM Akt inhibitor MK2206. Cells were fixed and stained for immunofluorescence (as described in the text) using mouse monoclonal Nrf-2 antibody A-10 (Santa Cruz Biotechnology) and Alexa-Fluor 488 conjugated donkey anti-mouse. Antibody A-10 was previously used to detect Nrf-2 translocation in A549 cells in response to bacterial pyocyanin (25). Images were taken at 40x (LWD 0.6 NA; scale bar is 20 μm) and nuclear fluorescence intensity was quantified by using automatic thresholding of the DAPI image in ImageJ to create regions of interest (ROIs) of the nuclei that were superimposed on the Nrf-2 channel. Images shown are identically processed. Note increase in nuclear Nrf-2 with with SC79 that was inhibited by MK2206. Bar graph shows results from independent experiments; each experiment is average of 3 fields of one well of a 24 plate. Significance in bar graph determined by one way ANOVA with Bonferroni posttest; **p<0.01. (b) Similar experiments were carried out after 18 hours in vehicle only (control), SC79, TNF-a (0.1 μg/ml) ± SC79, or flagellin (1 μg/ml) ± SC79. Note strong nuclear fluorescence of Nrf-2 only in the presence of SC79. Significance in bar graph determined by one way ANOVA with Bonferroni posttest; ##p<0.01 vs control and **p<0.01 between bracketed group. We hypothesize that increased nuclear Nrf-2 stimulated by SC79 is reduces IL-8 during TNF-a or flagellin stimulation. **Figure S10.** SC79 induces nuclear GFP-Nrf-2. A549s on 8-well chambered coverglass were transfected with GFP-Nrf-2 (Addgene # 21549; (26)) using 0.05 μg GFP-Nrf-2 DNA + 0.25 μg empty pcDNA3.0 vector per well to limit GFP-Nrf-2 overexpression. Higher levels of GFP-Nrf-2 cDNA lead to nuclear accumulation at baseline. (a-d) Living cells were imaged every 30 min over the course of 2 hours then once again at 4 hours. A programable motorized stage (Prior Scientific, Rockland MA) was used on an inverted Olympus (Tokyo, Japan) IX83 microscope with 40x (0.75 NA) objective and standard FITC filter set. Unstimulated cells (a) were compared with cells stimulated with SC79 (1 μg/ml; b) or SC79 + 5 μM Akt inhibitor MK2206 (c) or SC79 + 1 μM PI3K inhibitor LY29004 (d). Inhibitors were added to cells 30 min prior to the experiment. SC79 was added at time 0. Images shown are from 0, 0.5, 1, 1.5, 2, and 4 hours after stimulation. Not increase in bright nuclear fluorescence with SC79 alone. (e) Results were quantified by taking the total GFP-Nrf-2 cellular fluorescence (normalized to time 0) from 3 different experiments, each experiment imaging 2-8 GFP-Nrf-2 transfected cells. Significance at 4 hours was determined by one-way ANOVA with Dunnett’s posttest comparing all values to unstimulated control. Only SC79 alone was significantly different from control; **p<0.01. (f) In separate experiments, cells were imaged at 18 hours after no stimulation (control) or 18 hours 1 μg/ml SC79 and/or TNF-a. Note strong nuclear fluorescence in cells with SC79 vs control or TNF-a. Images are shown from one single experiment per condition, each experiment imaging 3 independent random fields. Images are representative of 3 experiments per condition. Together with immunofluorescence of endogenous Nrf-2 in the Figure S9, these experiments support that SC79 activation of Akt enhances nuclear Nrf-2 levels under unstimulated and TNF-a stimulated conditions. **Figure S11.** Akt activation protects airway cells against cadmium-induced airway barrier dysfunction. (a) 16HBE14o- cells were grown in ALI-cultures and were treated with cadmium ± SC79 for 24 h and TEER measurements were taken. Bars are means ± SEM; n=3 experiments. *p<0.05 (SC vs. cadmium 100μM) and ***p<0.001 (cadmium 100μM vs. cadmium +SC79) by ANOVA and Bonferroni posttest. (b) Cells were treated as in Fig. S8a and cell lysates were collected for Western blotting to detect ZO-1. Bars are means ± SEM; n=3 experiments. ***p<0.001 (SC vs. cadmium) and ***p<0.001 (cadmium 100μM + SC) by ANOVA and Bonferroni posttest. (c) Cells were treated with SC79 ± PI3K inhibitor (LY294002,10μg/ml) for 2 h and cell lysates were collected for Western blotting to detect ZO-1. Bars are means ± SEM; n=3 experiments. **p<0.001 (SC vs. SC+LY) by ANOVA and Bonferroni posttest.

## Data Availability

All data generated or analyzed during this study are included in this article and its additional files.

## Abbreviations

Akt: Protein kinase B
ALI: Air–liquid interface
CF: Cystic fibrosis
COPD: Chronic obstructive lung disease
DAF-FM: 4-Amino-5-methylamino-2′,7′-difluorofluorescein
eNOS: Endothelial nitric oxide synthase
FRET: Förster resonance energy transfer
HO-1: Heme oxygenase 1
IL-8: Interleukin-8
IL-17: Interleukin-17
iNOS: Inducible nitric oxide synthase
KEAP-1: Kelch-like ECH associated protein-1
L-NAME: L-*N*^G^-nitroarginine methyl ester
LY294002: PI3K inhibitor
NF-κB: Nuclear factor-kappa B
NO: Nitric oxide
NOS: Nitric oxide synthase
NQO-1: NAD(P)H quinone dehydrogenase 1
Nrf-2: Nuclear factor erythroid 2–related factor 2
PH: Pleckstrin homology
PI3K: Phosphoinositide-3-kinase
PKA: Protein kinase A
PKC: Protein kinase C
TEER: Transepithelial electrical resistance
TJ: Tight junctions
TLR: Toll-like receptor
TNF-α: Tumor necrosis factor-alpha
ZO-1/2: Zonula occludens 1/2
