# Supplementary material for: Small-molecule Akt-activation in airway cells induces NO production and reduces IL-8 transcription through Nrf-2
Source: Respir Res. 2021 Oct 19;22:267. doi: 10.1186/s12931-021-01865-y (PMC8525858; doi:10.1186/s12931-021-01865-y)
Supplement: Supplementary file 1 — Additional file 1: Figure S1. Co-localization and expression of glut1 and eNOS in A549 cells. IF was performed for GLUT1 (SLC2A1) and eNOS as described in the main text. (a) Secondary antibody only control, (b) glut-1 (cyan) and eNOs (magenta) localized largely to the plasma membrane, and (c) Line-scan showing colocalization of glut1 and eNOS. Scale bar is 10 μm. Figure S2. Inhibition of PI3K with 1μg/ml LY294002 reverses SC79-induced p-Akt. A549s were treated with SC79 (10μg/ml; DMSO only as control) or PI3K inhibitor LY294002 (1μg/ml) for 1 h before incubating with SC79 for 2 h. Cell lysates were collected for Western. Bars are means ± SEM; n = 3 experiments. (a) *p<0.05 (CTL vs. SC) and **p<0.01 (SC vs. SC+LY) by ANOVA and Bonferroni posttest. Figure S3. SC79-induced Akt activity, visualized by AktAR, is sustained over 18 hours. (a) AktAR is constructed from cyan fluorescent protein (CFP) variant cerulean and YFP-variant circularly permutated (cp)Venus with an E172 mutation surrounding a forkhead-associated domain (FHA1) used as the phosphorylated amino acid binding domain and the sequence surrounding Thr-24 of FOXO1 as an Akt substrate (10). (b) Phosphorylation by Akt causes a conformational shift that increases FRET between the CFP and YFP moieties that increases YFP emission and decreases CFP emission with CFP excitation. (c) Representative trace showing AktAR fluorescence changes. After transfection with AktAR (Lipofectamine 3000, 24 hrs., as described in the text), cells on chambered coverglass were imaged on an inverted microscope (Olympus, Tokyo Japan; 20x 0.8 NA objective) with motorized programmable stage (Prior Scientific, Rockland MA) and standard CFP/YFP emission filters in motorized filter wheels (Lambda LS, Sutter Instruments, Novato California). CFP and YFP emission (both with CFP excitation) was collected every 2 min for 3 hours. Cells were stimulated with 1 μg/ml SC-79, 50 ng/ml IGF-1, 0.1 μg/ml TNFa, or buffer alone after 8 min. After overnight [file 12931_2021_1865_MOESM1_ESM.pdf]

Additional Methods

Reagents and solutions

Anti-ZO-1 (#40-2200) and XTT (#6493) were purchased from ThermoFisher (Waltham, MA, USA). L- and D-N^G-nitroarginine methyl ester (L-NAME and D-NAME), SC79 (#14972), LY294002 (#70920), ML385 (#21114) were purchased from Cayman Chemical (Ann Arbor, MI, USA). Glut-1 (#15309) was purchased from Abcam, Inc. (Cambridge, MA, USA). 4-amino-5-methylamino-2′,7′-difluorofluorescein diacetate (DAF-FM diacetate) was from Life Technologies (Carlsbad, California, USA). Anti-Nrf-2 antibody A10 (sc-365949) was purchased from Santa Cruz Biotechnology (Dallas, TX, USA). Unless indicated below, all other reagents were from Sigma Aldrich (St. Louis, MO, USA).

Primary bronchial, primary nasal, and ALI cultures

Patient tissue was obtained with written informed consent and approval from the University of Pennsylvania Institutional Review Board (protocol #800614). Tissue acquisition was carried out in accordance with The University of Pennsylvania guidelines regarding use of residual clinical material, using tissue from patients ≥18 years of age undergoing surgery for sinonasal disease (CRS) or other procedures (e.g. trans-nasal approaches to the skull base). Written informed consent was obtained in accordance with the U.S. Department of Health and Human Services Code of federal regulation Title 45 CFR 46.116 and the Declaration of Helsinki. Adult patients undergoing sinonasal surgery were recruited from the Department of Otorhinolaryngology at the University of Pennsylvania and the Philadelphia Veterans Affairs Medical Center. Patients with a history of genetic disease (e.g. cystic fibrosis, primary ciliary dyskinesia), immunodeficiencies, or use of oral corticosteroids or antibiotics within 1 month of surgery were excluded from the study. Sinonasal tissue specimens were obtained from residual clinical material following sinonasal surgery and were transported to the laboratory in saline placed on ice. Human primary nasal epithelial cells were obtained through enzymatic dissociation of human sinonasal tissue (1, 2). Primary bronchial epithelial cells were obtained from Lonza (Cat. # CC-2540S; Walkerville, MD, USA) and cultured as described previously (1). Both primary bronchial and nasal cells were grown to confluence in 10 cm tissue culture dishes with PneumaCult-Ex Plus culture medium (Cat. #05040; STEMCELL Technologies, Vancouver, BC, Canada).

To obtain primary nasal ALI cultures, cells were plated, and 400 µl of growth medium were added. The following day, the culture medium in the apical compartment was aspirated and the medium in the basal compartment was replaced with 400 µl of PneumaCult-ALI culture medium (Cat. #05001; STEMCELL

Technologies, Vancouver, BC, Canada) to allow for mucociliary differentiation. The culture medium in the basal compartment was changed every other day for 28 days until cultures achieved differentiation.

Air-liquid interface (ALI) cultures and transepithelial electrical resistance (TEER) measurements

16HBE14o- cells were cultured on 12-well transwell cell culture inserts (Greiner Bio-One, Monroe, NC, USA) fitted with 0.4-µm-pore-size membranes and were grown submerged for one week before exposing the apical side to air for an additional two weeks. While they do not form multiple cell types and a pseudostratified epithelial layer, 16HBEs are a non-cancerous line that mimics many phenotypes of primary cells including TEER formation (3, 4), and have been extensively used for epithelial barrier studies (5). At the time of the experiment, cells were treated from both basolateral and apical sides. Cadmium (100 µM) ± SC79 (10 µg/ml) was used to model epithelial injury after 24 h of treatment. TEER measurements were taken using an epithelial volt-Ohm meter, ($EVOM^2$, World Precision Instruments, Sarasota, FL, USA). Cell lysates were collected to measure ZO-1 by Western blotting.

H441-distal lung epithelial cell culture

H441 cells (ATCC HTB-174; Manassas, VA, USA) were cultured in MEM with Earle's salts (Gibco, Gaithersburg, MD, USA). When 75% confluence was reached, cells were transferred to 24- well plates for NO measurements. H441 cells were shown previously to express eNOS (6, 7) and acutely produce NO in response to agonists such as 17-β-estradiol (8).

Macrophage (MΦ) culture

Monocytes were isolated from healthy apheresis donors by the Human Immunology Core (University of Pennsylvania, Philadelphia, PA) with institutional review board approval by RosetteSep™ human monocyte enrichment cocktail (STEMCELL Technologies, Vancouver, BC, Canada) and were grown as described previously (9). Monocytes were grown for 10-days and were differentiated by 10-days adherence culture in RPMI-2650 media containing 10% human serum and 1x pen/strep antibiotic mix.

NO imaging

Stock DAF-FM diacetate was dissolved at 1 mM in DMSO. MΦs and H441 cells were grown in 8-well chamber slides. On the day of the experiment, cells were loaded with 5 µM cell permeant DAF-FM diacetate diluted in HEPES-buffered HBSS for 60 min at room temperature. The cells were washed 3X with HBSS to remove unloaded DAF-FM and were stimulated with SC79 (1-10µg/ml). DAF-FM fluorescence

images were acquired at 12-second intervals (main text figure) or 30 sec intervals (additional figures) using an IX83 inverted microscope (Olympus, Tokyo, Japan, USA) equipped with a 20x 0.8 NA objective, FITC filter set (Semrock), and MetaFluor Software (Molecular Devices, Sunnyvale, CA, USA) as described previously (1, 2). Analysis of background-subtracted fluorescence intensity changes were made by normalizing fluorescence to fluorescence at time 0 (F/F_o)

XTT assay

Cell viability and cytotoxicity were evaluated using XTT (2,3-bis-(2-methoxy-4-nitro-5-sulfophenyl)-2Htetrazolium-5-carboxanilide) to measure proliferation based on the metabolic activity of the cells. Briefly, A549 cells were grown in 48-well culture plates containing 200µl of growth medium. Next, cells were treated with DMSO (CTL) or SC79 (2.5µg/ml) for 24 h. After, 100µl of XTT/PMS (Phenazine methosulfate) solution was added to each well for an additional 1 h. Cell viability was measured at 475nm and 660nm. All data for XTT assays were obtained by Tecan (Männedorf, Switzerland) Spark 10M.

Additional Figures

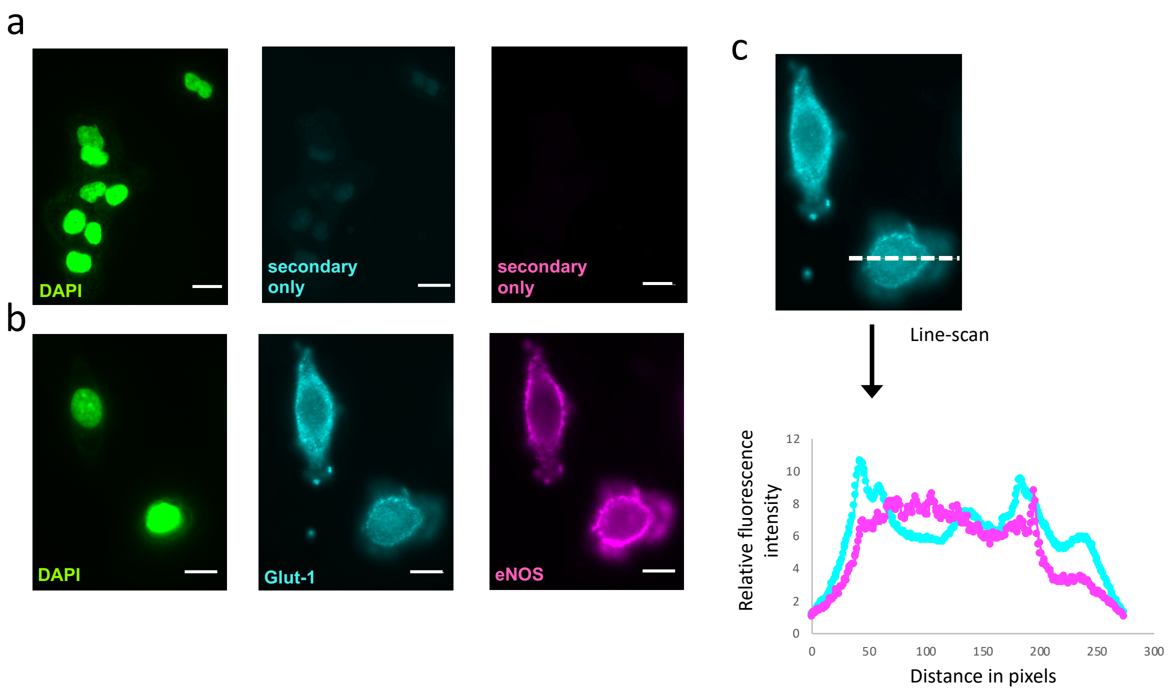

Figure S1. Co-localization and expression of glut1 and eNOS in A549 cells. IF was performed for GLUT1 (SLC2A1) and eNOS as described in the main text. **(a)** Secondary antibody only control, **(b)** glut-1 (cyan) and eNOs (magenta) localized largely to the plasma membrane, and **(c)** Line-scan showing colocalization of glut1 and eNOS. Scale bar is 10 µm.

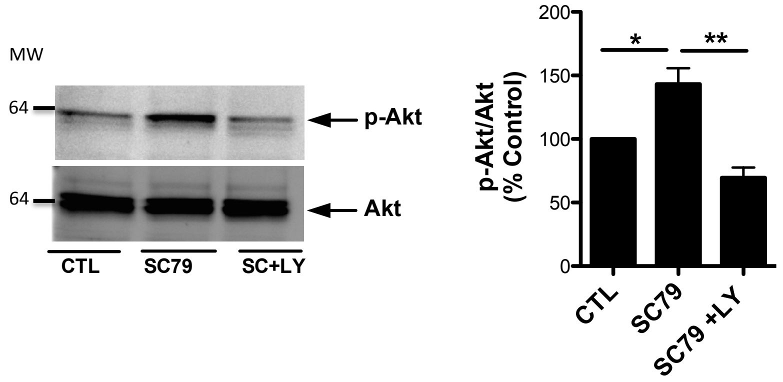

Figure S2. Inhibition of PI3K with 1µg/ml LY294002 reverses SC79-induced p-Akt. A549s were treated with SC79 (10µg/ml; DMSO only as control) or PI3K inhibitor LY294002 (1µg/ml) for 1 h before incubating with SC79 for 2 h. Cell lysates were collected for Western. Bars are means ± SEM; n = 3 experiments. **(a)** *p<0.05 (CTL vs. SC) and **p<0.01 (SC vs. SC+LY) by ANOVA and Bonferroni posttest.

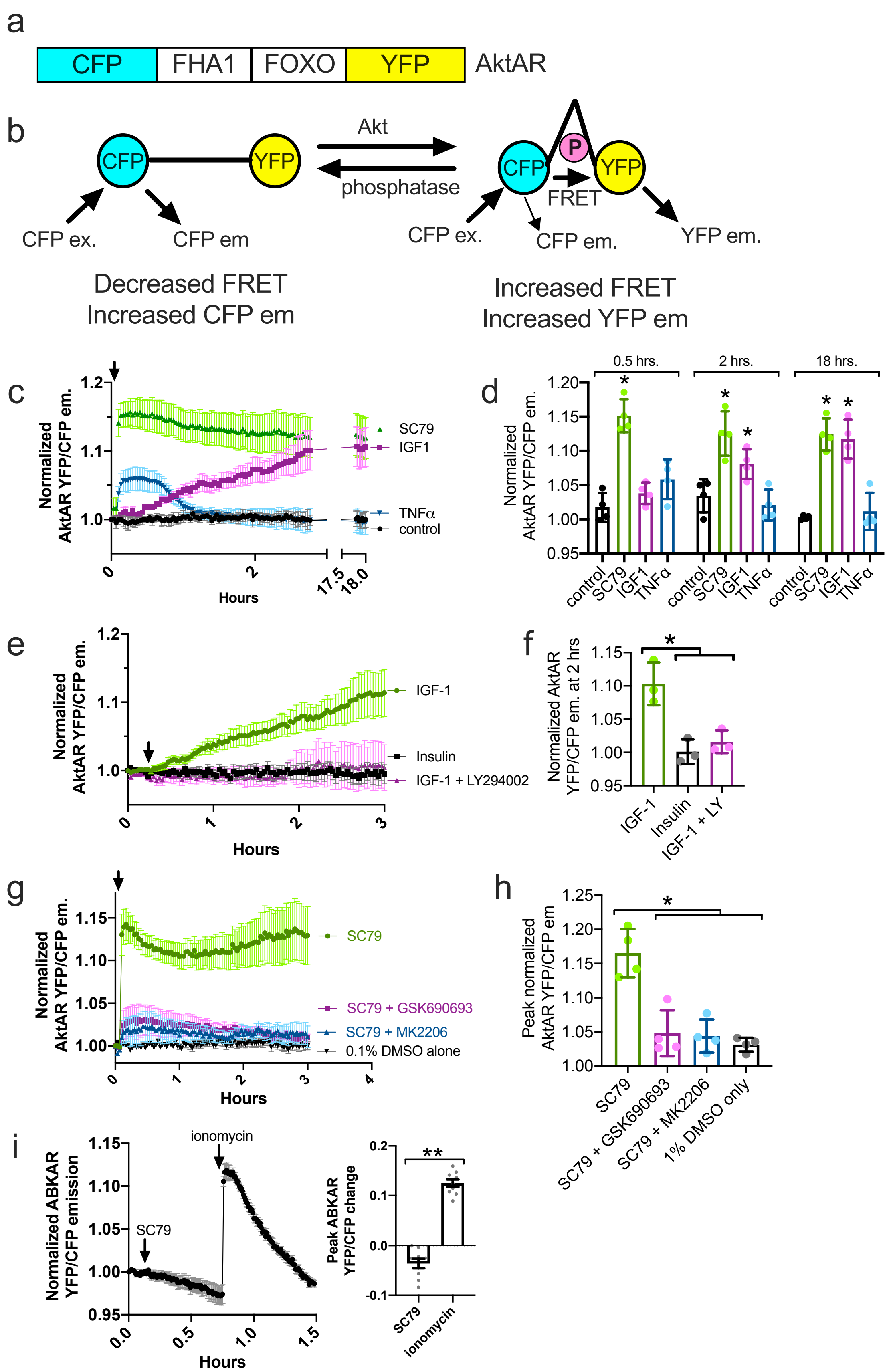
a
CFP
FHA1
FOXO
YFP
AktAR
b
CFP
YFP
Akt
phosphatase
P
FRET
CFP ex.
CFP em
CFP em.
YFP em.
Decreased FRET
Increased CFP em
Increased FRET
Increased YFP em
c
Normalized AktAR YFP/CFP em.
Hours
SC79
IGF1
TNFα
control
d
Normalized AktAR YFP/CFP em.
0.5 hrs.
2 hrs.
18 hrs.
control
SC79
IGF1
TNFα
e
Normalized AktAR YFP/CFP em.
IGF-1
Insulin
IGF-1 + LY294002
Hours
f
Normalized AktAR YFP/CFP em. at 2 hrs
IGF-1
Insulin
IGF-1 + LY
g
Normalized AktAR YFP/CFP em.
SC79
SC79 + GSK690693
SC79 + MK2206
0.1% DMSO alone
Hours
h
Peak normalized AktAR YFP/CFP em
SC79
SC79 + GSK690693
SC79 + MK2206
1% DMSO only
i
Normalized ABKAR YFP/CFP emission
SC79
ionomycin
Hours
Peak ABKAR YFP/CFP change

Figure S3. SC79-induced Akt activity, visualized by AktAR, is sustained over 18 hours. **(a)** AktAR is constructed from cyan fluorescent protein (CFP) variant cerulean and YFP-variant circularly permutated (cp)Venus with an E172 mutation surrounding a forkhead-associated domain (FHA1) used as the phosphorylated amino acid binding domain and the sequence surrounding Thr-24 of FOXO1 as an Akt substrate (10). **(b)** Phosphorylation by Akt causes a conformational shift that increases FRET between the CFP and YFP moieties that increases YFP emission and decreases CFP emission with CFP excitation. **(c)** Representative trace showing AktAR fluorescence changes. After transfection with AktAR (Lipofectamine 3000, 24 hrs., as described in the text), cells on chambered coverglass were imaged on an inverted microscope (Olympus, Tokyo Japan; 20x 0.8 NA objective) with motorized programmable stage (Prior Scientific, Rockland MA) and standard CFP/YFP emission filters in motorized filter wheels (Lambda LS, Sutter Instruments, Novato California). CFP and YFP emission (both with CFP excitation) was collected every 2 min for 3 hours. Cells were stimulated with 1 μg/ml SC-79, 50 ng/ml IGF-1, 0.1 μg/ml TNFα, or buffer alone after 8 min. After overnight incubation on the stage of the microscope, cells were also imaged at 17.5-18 hours (note break in axis). Traces suggest sustained Akt activity with SC-79 or IGF-1 but not TNFα or control (media only). **(d)** Bar graph of n = 4 experiments as in *c*. Data points show AktAR YFP/CFP em from individual experiments at 3 time points: 0.5, 2, and 18 hrs. Significance by one-way ANOVA with Dunnett's posttest comparing each stimulated conditions (SC79, IGF1, or TNFα) to control unstimulated condition at each time point. Bars are mean ± SEM; *$p<0.01$. **(e)** Trace showing increase in AktAR YFP/CFP emission with 10 nM IGF-1 but not 10 nM insulin. IGF-1 YFP/CFP emission increase was also blocked by 1 μg/ml LY294002 (PI3K inhibitor). This fits previous studies showing IGF-1 is a more potent activator of Akt signaling than insulin in A549s (11). **(f)** Bar graph showing independent experiments (n = 3) and mean ± SEM from experiments as in *e*. YFP/CFP emission at 2 hours is shown. Significance by one-way ANOVA with Dunnett's posttest comparing all values to IGF-1. **(g)** Representative traces showing AktAR YFP/CFP emission during SC79 stimulation with Akt inhibitors GSK690693 (1 μM) or MK2206 (5 μM). DMSO alone (0.1% was used as vehicle control. SC79 increased AktAR YFP/CFP emission, but this was reduced by Akt inhibitors. DMSO alone had no effect. **(h)** Bar graph showing mean ± SEM and individual values from experiments as in g. Peak AktAR YFP/CFP em is shown. Significance by one-way ANOVA comparing all values to SC79 (Dunnett's posttest); *$p<0.01$. Together, these data support that SC79 induces sustained Akt activation in A549s. **(i-j)** Representative trace (i) and bar graph showing change in ABKAR, a FRET reporter of AMP-activated protein kinase (AMPK) activity. AMPK is negatively regulated by Akt phosphorylation that prevents the accessibility of LKB1 or CAMMK to the activating T172 AMPK phosphorylation site (12-15) we see a reduction in YFP/CFP emission (reflecting AMPK activity) with SC79 but an increase in YFP/CFP emission (reflecting increased AMPK activity) with ionomycin, which was used in the original ABKAR paper (16) to activate AMPK by CaMKKβ (17). Significance in j by Student's t test; **$p<0.01$

Additional File 1: Gopallawa et al.
Small-molecule Akt-activation in airway cells induces NO production and reduces IL-8 transcription through Nrf-2

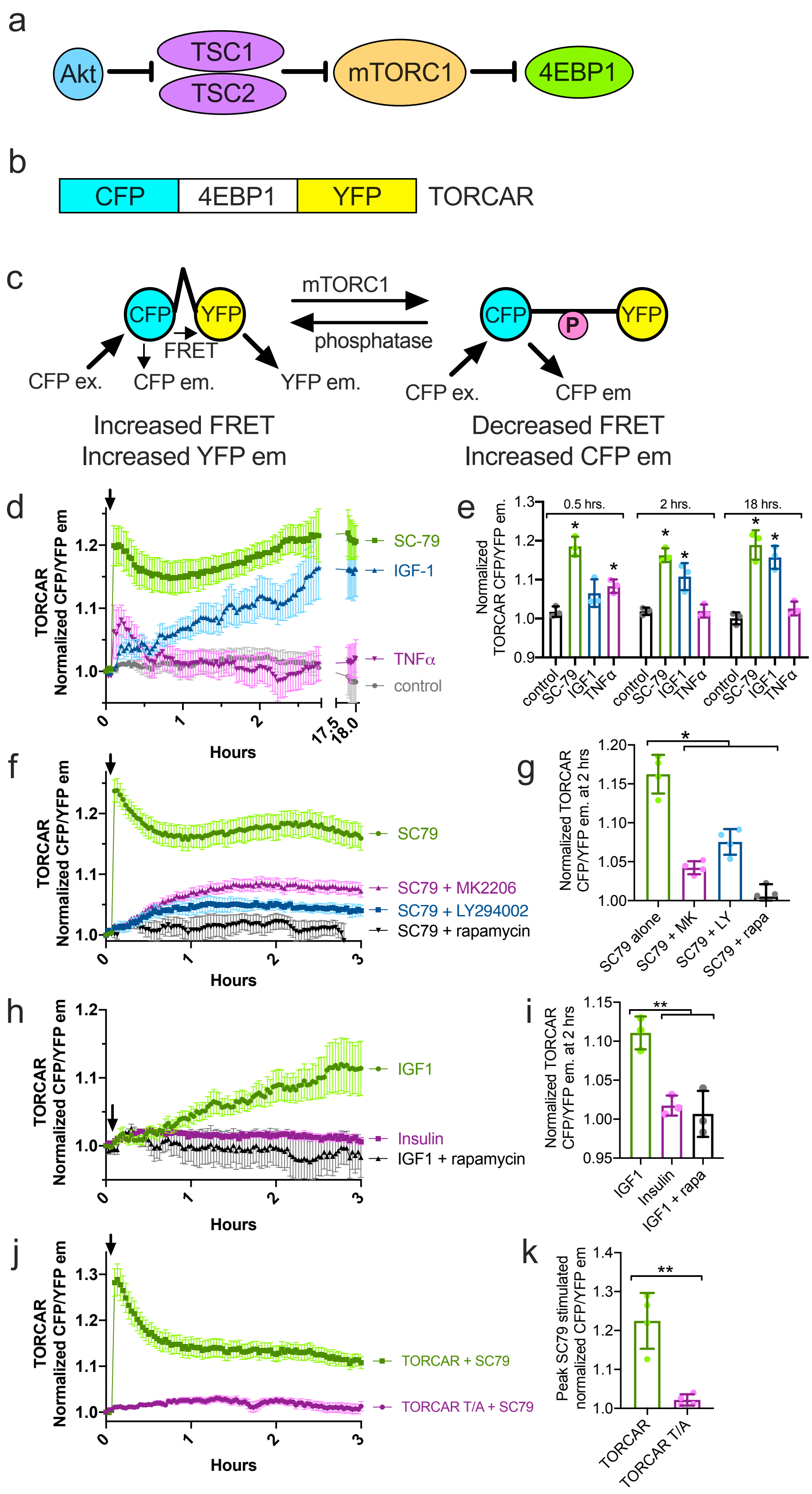

Figure S4. SC79 activates mTORC1 via Akt in A549 cells. **(a)** Activation of Akt results in phosphorylation of TSC2 at S939, S981, and S1462 recruit 14-3-3 to TSC2 and disrupt the TSC1/TSC2 dimer, allowing increased mTORC1 activity (18-20). In other words, Akt inhibition of TSC1/TSC2 dimerization reduces TSC1/TSC2 inhibition of mTORC1. Thus, Akt activation can enhance mTORC1 activity. **(b)** We visualized mTORC1 activity using fluorescent biosensor TORCAR (21, 22); Addgene plasmid #64927) transfected into A549 cells. TORCAR contains 4EBP1, a mTORC1 phosphorylation substrate, flanked by cyan fluorescent protein (CFP) variant cerulean and yellow fluorescent protein (YFP) variant YPet. **(c)** Phosphorylation of the 4EBP1 in TORCAR at T37 and T46 results in a conformation shift in the TORCAR protein that decreases FRET between the CFP and YFP. This increases CFP emission and reduces YFP emission during CFP excitation. **(d)** After transfection with TORCAR (Lipofectamine 3000, 24 hrs., as described in the text), cells on chambered coverglass were imaged on an inverted microscope (Olympus, Tokyo Japan; 20x 0.8 NA objective) with motorized programmable stage (Prior Scientific, Rockland MA) and standard CFP/YFP emission filters in motorized filter wheels (Lambda LS, Sutter Instruments, Novato California). CFP and YFP emission (both with CFP excitation) was collected every 2 min for 3 hours. After overnight incubation on the stage of the microscope, cells were also imaged at 17.5-18 hours (note break in axis). Cells were stimulated with 1 μg/ml SC-79, 50 ng/ml IGF-1, 0.1 μg/ml TNFα, or buffer alone after 8 min. Note sustained mTORC1 activity with SC-79 and IGF-1 but not TNFα. However, onset of mTORC1 activity was more rapid with SC-79 compared with IGF-1. Traces shown are from the same single experiment where 8 transfected cells were imaged per condition. Each condition was from a separate well of the same chamber slide. **(e)** Bar graph of n = 3 experiments as in (d). Data points show TORCAR CFP/YFP em from individual experiments at 3 time points: 0.5, 2, and 18 hrs. Significance by one-way ANOVA with Dunnett's posttest comparing each stimulated conditions (SC79, IGF1, or TNFα) to control unstimulated condition at each time point. Bars are mean ± SEM; *p<0.01. **(f)** Representative experiment as in *d* but with SC79 ± Akt inhibitor MK2206 (5 μM), PI3K inhibitor LY294002 (1 μM), or mTOR inhibitor rapamycin (0.5 μM) over 3 hrs. Cells were pretreated with inhibitors for 20 min before the addition of 1 μg/ml SC79. **(g)** Bar graph showing data from 3 independent experiments as in *f* for TORCAR CFP/YFP emission at 2 hours. TORCAR CFP/YFP emission ratio was reduced in the presence of Akt, PI3K, or mTOR inhibitor. Significance by one way ANOVA with Dunnett's posttest comparing all values to SC79 alone. **(h)** Representative experiment showing TORCAR CFP/YFP emission during stimulation with 10 nM insulin or 10 nM IGF1 ± rapamycin. Note increase in TORCAR CFP/YFP emission with IGF1 but not insulin and inhibition with rapamycin. **(i)** Bar graph showing independent experiments and mean ± SEM from experiments as in *h*. Significance by one-way ANOVA with Dunnett's posttest comparing all values to IGF-1; **p<0.01. **(j)** Representative experiment showing CFP/YFP emission during SC79 stimulation in cells transfected with TORCAR or TORCAR T/A (Addgene plasmid #64928), which has mTORC1 phosphorylation sites mutated. Note increase in TORCAR but not TORCAR T/A fluorescence, showing that the changes observed depend on phosphorylation of this site, fitting with the rapamycin data above. **(k)** Bar graph of independent experiments as in j showing individual experiments and mean ± SEM. Significance by Student's t-test; ** p<0.01. Together, these studies confirm SC79 induces sustained activation of Akt by showing downstream sustained activation of mTORC1 in A549s.

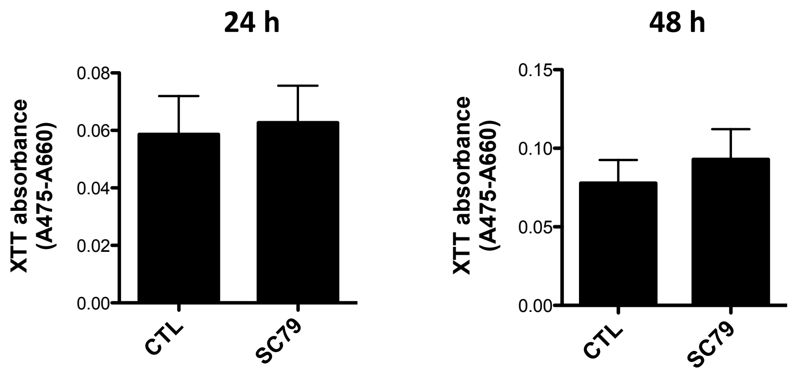

Figure S5. SC79 does not reduce cell metabolism at 24 h and 48 h. A549 cells were treated with SC79 (2.5μg/ml; DMSO only as control). After 24 or 48 hours, 100μl of XTT/PMS solution was added to each well for 1 h and the absorbance was measured at a wavelength of 450 nm and background absorbance was measured at 660 nm. Background absorbance was subtracted from signal absorbance to gain normalized values. Bars are means ± SEM; n = 3. The two groups were not significantly different by Student's t test.

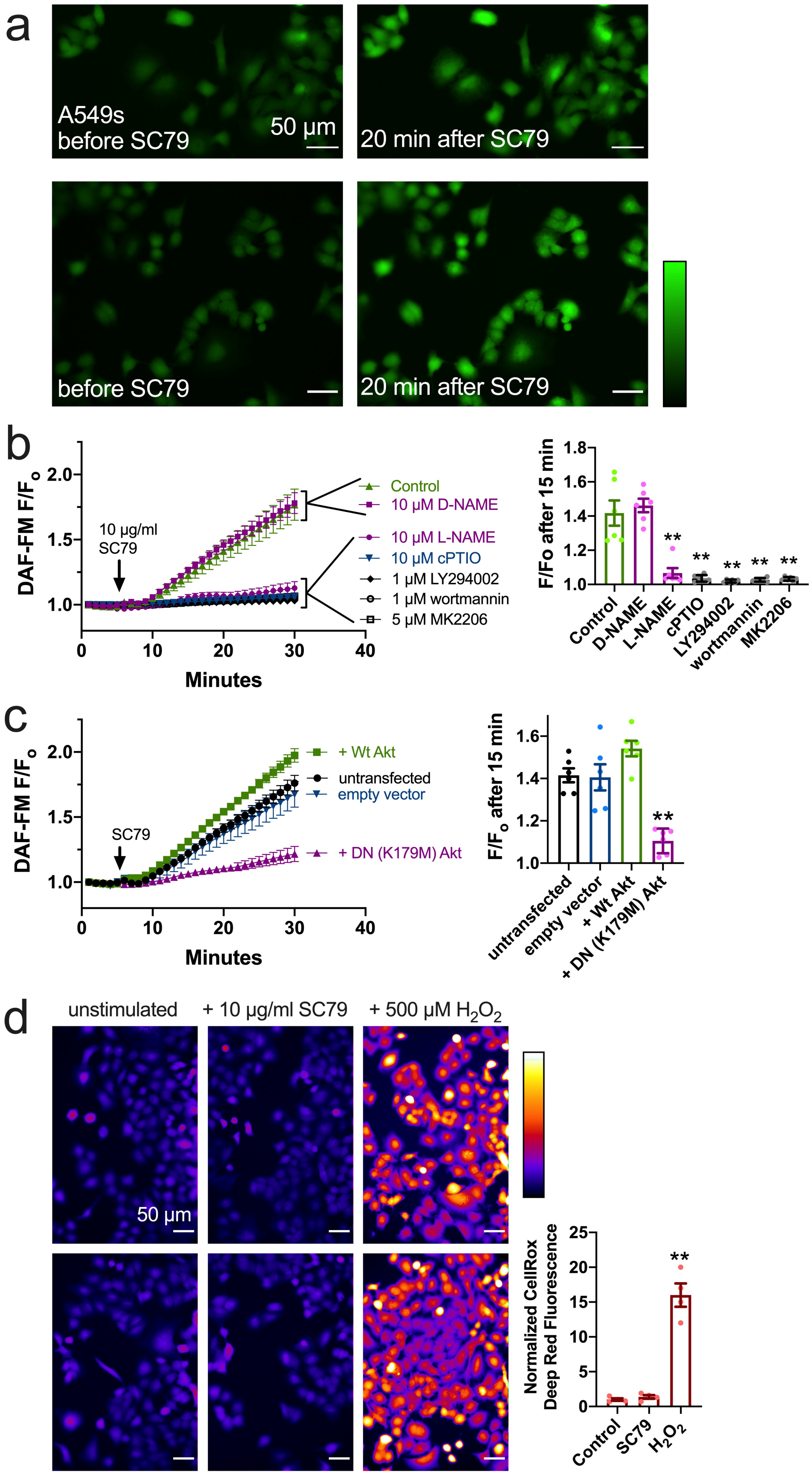
a
A549s
before SC79
50 µm
20 min after SC79
before SC79
20 min after SC79
b
DAF-FM F/Fo
10 µg/ml
SC79
Minutes
Control
10 µM D-NAME
10 µM L-NAME
10 µM cPTIO
1 µM LY294002
1 µM wortmannin
5 µM MK2206
F/Fo after 15 min
Control
D-NAME
L-NAME
cPTIO
LY294002
wortmannin
MK2206
c
DAF-FM F/Fo
SC79
Minutes
+ Wt Akt
untransfected
empty vector
+ DN (K179M) Akt
F/Fo after 15 min
untransfected
empty vector
+ Wt Akt
+ DN (K179M) Akt
d
unstimulated
+ 10 µg/ml SC79
+ 500 µM H2O2
50 µm
Normalized CellRox Deep Red Fluorescence
Control
SC79
H2O2

Figure S6. SC79 induced DAF-FM increases reflect Akt-dependent NO production in A549 cells. (a) Images of A549 cells before and after SC79 (1 µg/ml) stimulation. Note no overt changes in cell morphology after SC79 stimulation. DAF-FM images were taken at 20x (0.8 NA objective) using standard FITC excitation filters as described in the methods. **(b)** Left shows DAF-FM traces from separate individual experiments (mean ± SEM of 10-20 cells) during stimulation with SC79 in the absence of inhibitors (control) or after 45 min pretreatment with 10 µM L-NAME (nitric oxide synthase inhibitor) or D-NAME (inactive control). Note increase in DAF-FM fluorescence in D-NAME treated cells is comparable to control while increase in L-NAME treated cells is significantly blunted, supporting that DAF-FM changes reflect NOS activity. DAF-FM fluorescence increases were also blocked in the presence of 10 µM cPTIO (NO scavenger, added at the beginning of the experiment), further supporting that DAF-FM changes reflect NO production. DAF-FM fluorescence increase was also reduced in the presence of PI3K inhibitors LY294002 or wortmannin as well as Akt inhibitor MK2206. Together, these data suggest that DAF-FM changes during SC79 stimulation reflect Akt-induction of NOS activity. Right shows the bar graph of data points (DAF-FM fluorescence after 15 min) from individual experiments (n = 4-6 per condition). Bars are mean ± SEM. Control is SC79 alone. Significance by one-way ANOVA with Dunnett's posttest comparing all values to SC79 alone (control); ** $p<0.01$. **(c)** A549 cell were untransfected or transfected with empty vector (pcDNA3.0) or pcDNA containing Wt or dominant negative K179M Akt (Addgene plasmid # Plasmid #16243 or #734058; (23, 24)). Left shows traces from individual separate DAF-FM experiments (10-20 cells imaged per condition). NO production was reduced with K179M Akt expression. Right shows data points from individual experiments (n = 6 per condition). Significance by one-way ANOVA with Dunnett's posttest comparing all values to untransfected control; ** $p<0.01$. This further supports that increase in DAF-FM observed with SC79 are dependent on Akt signaling. **(d)** A549s were similarly loaded with CellRox Deep Red (ThermoFisher Scientific; general reactive oxygen species-sensitive dye) for 30 min in the presence of SC79 or H_2O_2 (positive control to induce oxidative stress). No change in Cell Rox Deep Red fluorescence was observed with SC79, further supporting that DAF-FM changes above reflect NO and not another oxidative species. Images are from representative experiments, taken using 20x (0.8 NA) objective and standard Cy5 excitation and emission filters. Bar graph shows mean ± SEM from individual independent experiments (n = 4 each). Significance determined by one way ANOVA with Dunnett's posttest comparing all values to control (unstimulated); ** $p<0.01$.

Additional File 1: Gopallawa et al.
Small-molecule Akt-activation in airway cells induces NO production and reduces IL-8 transcription through Nrf-2

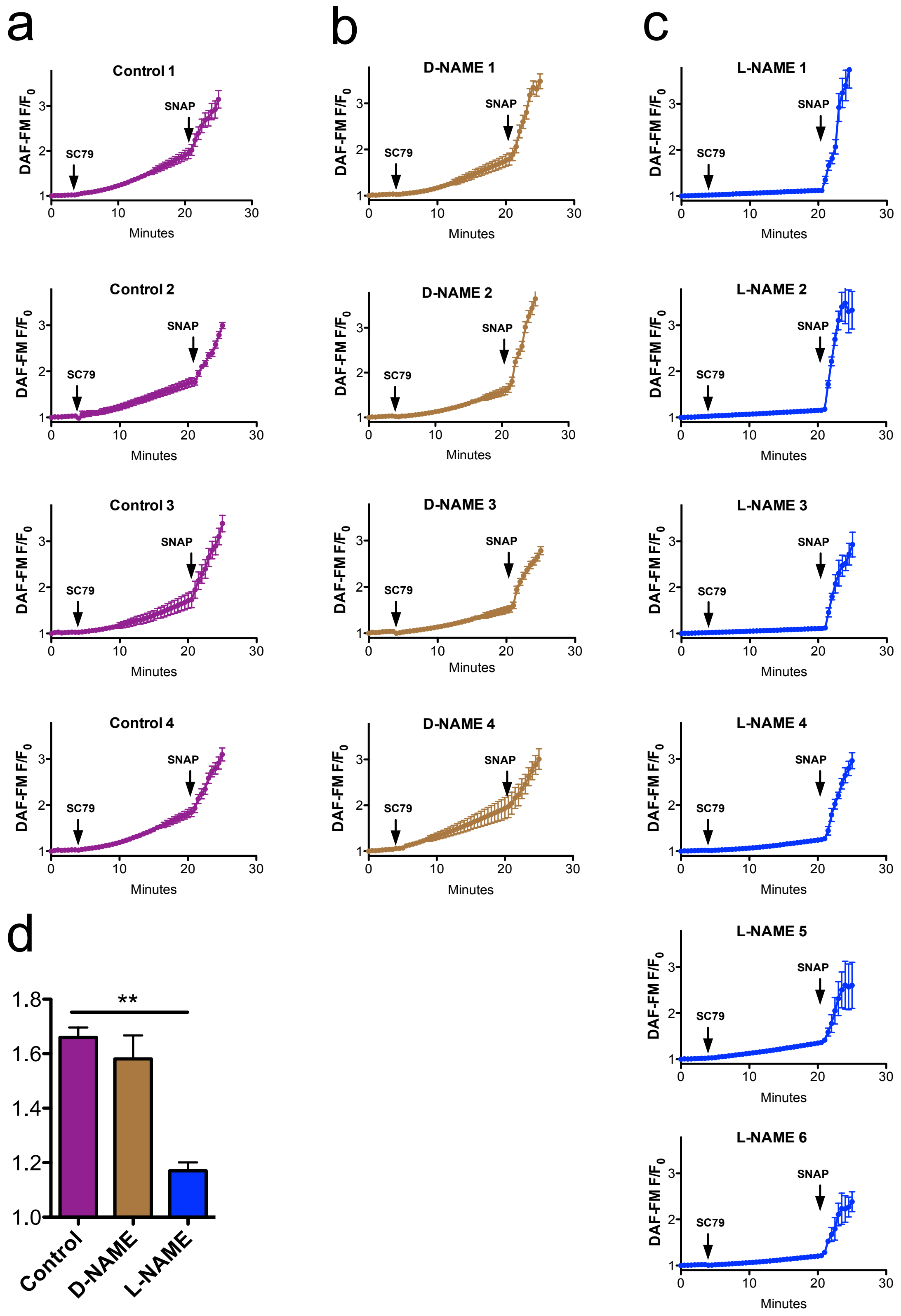

Figure S7. SC79 increased NO through eNOS in human distal lung epithelial cell-line H441. Cells were grown in 24-well chamber slides and were loaded with DAF-FM (5 μM) for 60 min before stimulating with SC79 (10 μg/ml). **(a)** Control, **(b)** D-NAME, inactive control for L-NAME (10 μM) and, **(c)** eNOS inhibitor L-NAME (10 μM). DAF-FM intensities were normalized at 15 min. Non-specific NO donor S-nitroso-N-acetyl-D, L-penicillamine (SNAP, 10 μM) was added at the end as a positive control. **(d)** Bars are means ± SEM; n ≥ 3 experiments. ***p*<0.01 (Control vs. L-NAME) by ANOVA and Bonferroni posttest.

Additional File 1: Gopallawa et al.
Small-molecule Akt-activation in airway cells induces NO production and reduces IL-8 transcription through Nrf-2

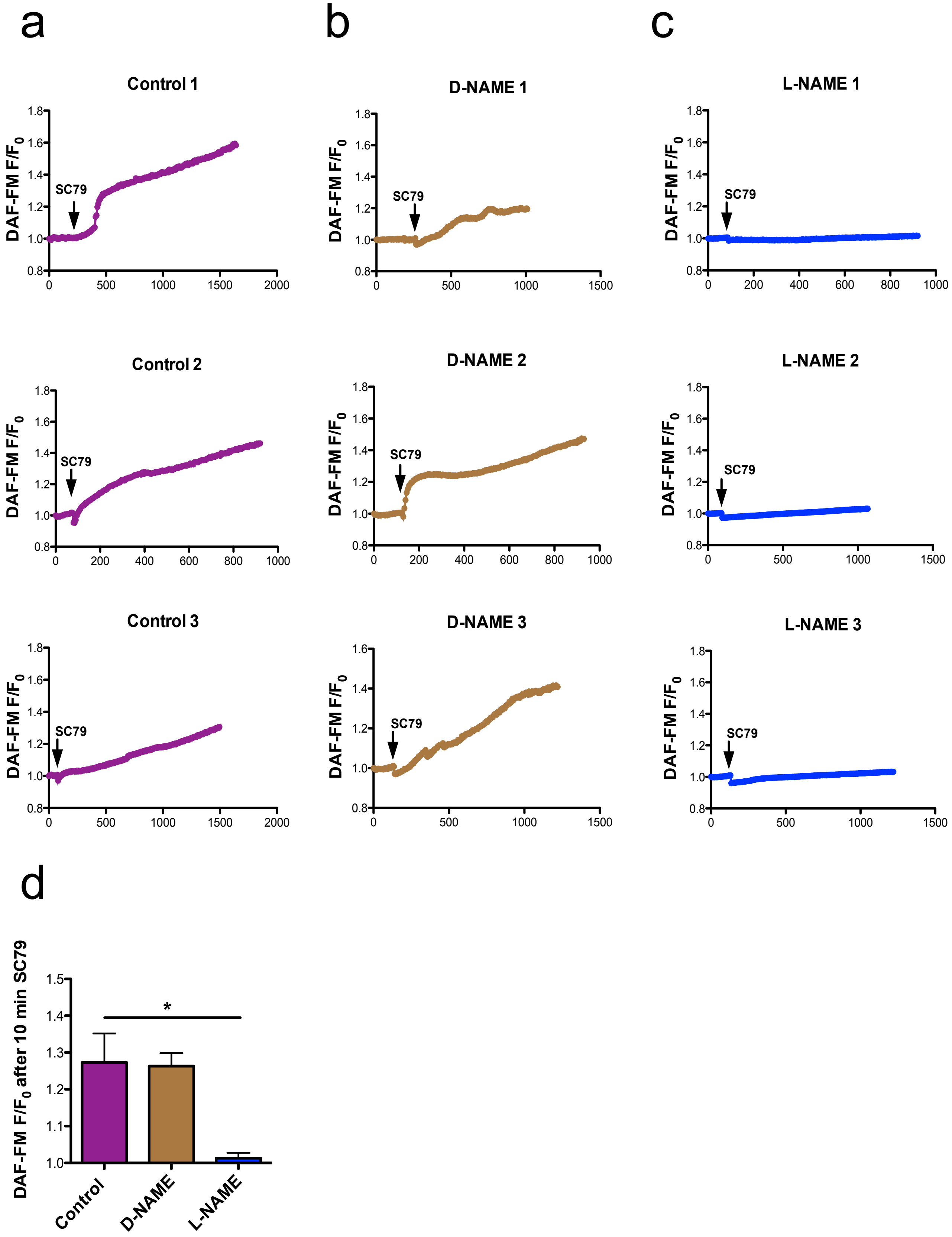

Figure S8. Induction of NO via eNOS in human primary MΦs with SC79. MΦs were loaded with DAF-FM and were stimulated with SC79 (10µg/ml). **(a)** Control, **(b)** D-NAME, inactive control for L-NAME (10 µM) and, **(c)** eNOS inhibitor L-NAME (10 µM). DAF-FM intensities were normalized at 10 min. **(d)** Bars are means ± SEM; n=3 experiments. *$p<0.05$ (Control vs. L-NAME) by ANOVA and Bonferroni posttest.

Additional File 1: Gopallawa et al.
Small-molecule Akt-activation in airway cells induces NO production and reduces IL-8 transcription through Nrf-2

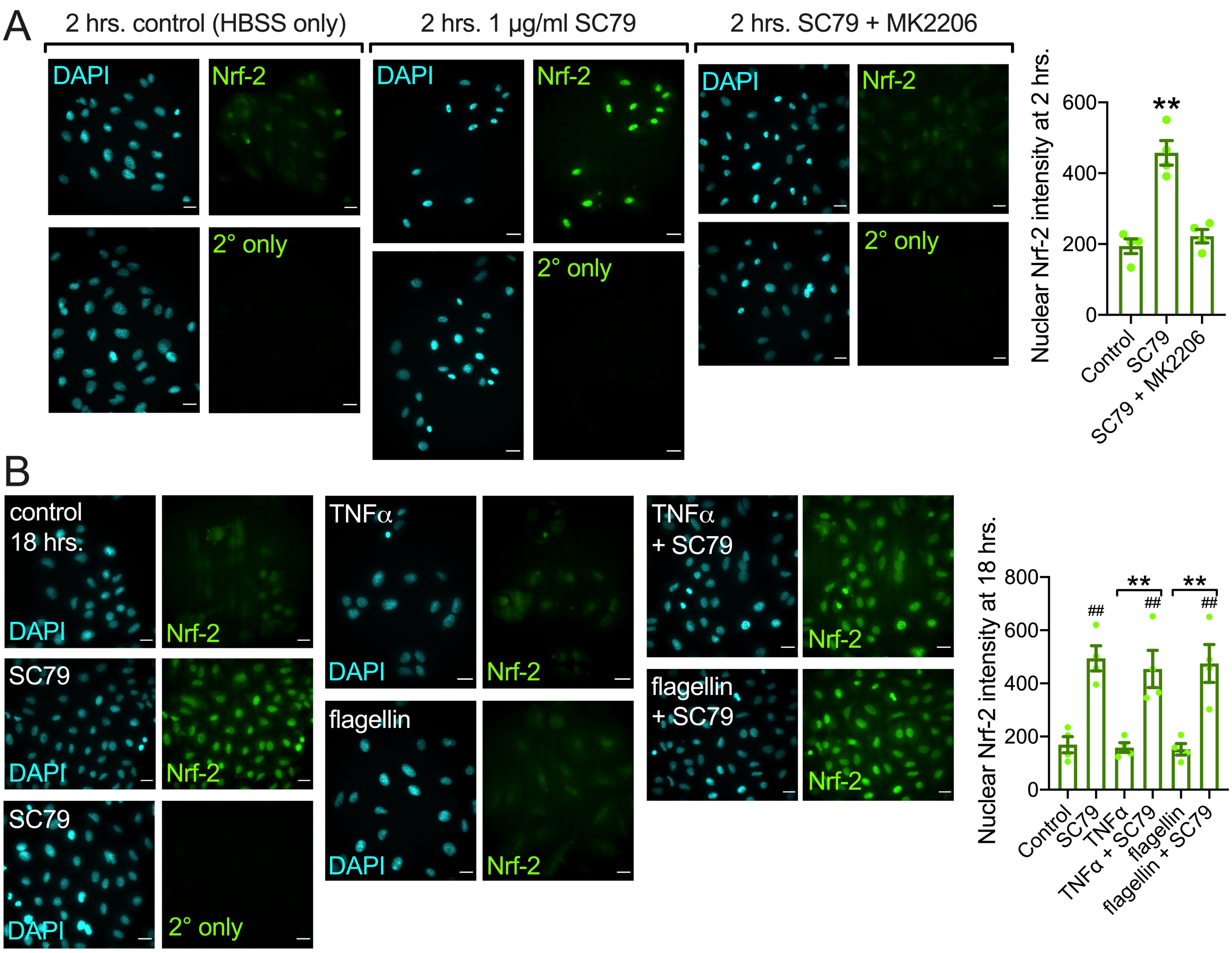

Figure S9. SC79 induces nuclear Nrf-2 accumulation by immunofluorescence of endogenous Nrf-2. **(a)** A549 cells were grown in plastic 24 well plates and incubated for 2 hours in Hank's balanced salt solution (HBSS) with 20 mM HEPES in the presence of vehicle only (0.1% DMSO; control), 1 µg/ml SC-79, or 1 µM SC79 + 5 µM Akt inhibitor MK2206. Cells were fixed and stained for immunofluorescence (as described in the text) using mouse monoclonal Nrf-2 antibody A-10 (Santa Cruz Biotechnology) and Alexa-Fluor 488 conjugated donkey anti-mouse. Antibody A-10 was previously used to detect Nrf-2 translocation in A549 cells in response to bacterial pyocyanin (25). Images were taken at 40x (LWD 0.6 NA; scale bar is 20 µm) and nuclear fluorescence intensity was quantified by using automatic thresholding of the DAPI image in ImageJ to create regions of interest (ROIs) of the nuclei that were superimposed on the Nrf-2 channel. Images shown are identically processed. Note increase in nuclear Nrf-2 with with SC79 that was inhibited by MK2206. Bar graph shows results from independent experiments; each experiment is average of 3 fields of one well of a 24 plate. Significance in bar graph determined by one way ANOVA with Bonferroni posttest; $^{**}p<0.01$. (b) Similar experiments were carried out after 18 hours in vehicle only (control), SC79, TNF-α (0.1 µg/ml) ± SC79, or flagellin (1 µg/ml) ± SC79. Note strong nuclear fluorescence of Nrf-2 only in the presence of SC79. Significance in bar graph determined by one way ANOVA with Bonferroni posttest; ##p<0.01 vs control and **p<0.01 between bracketed group. We hypothesize that increased nuclear Nrf-2 stimulated by SC79 is reduces IL-8 during TNF-α or flagellin stimulation.

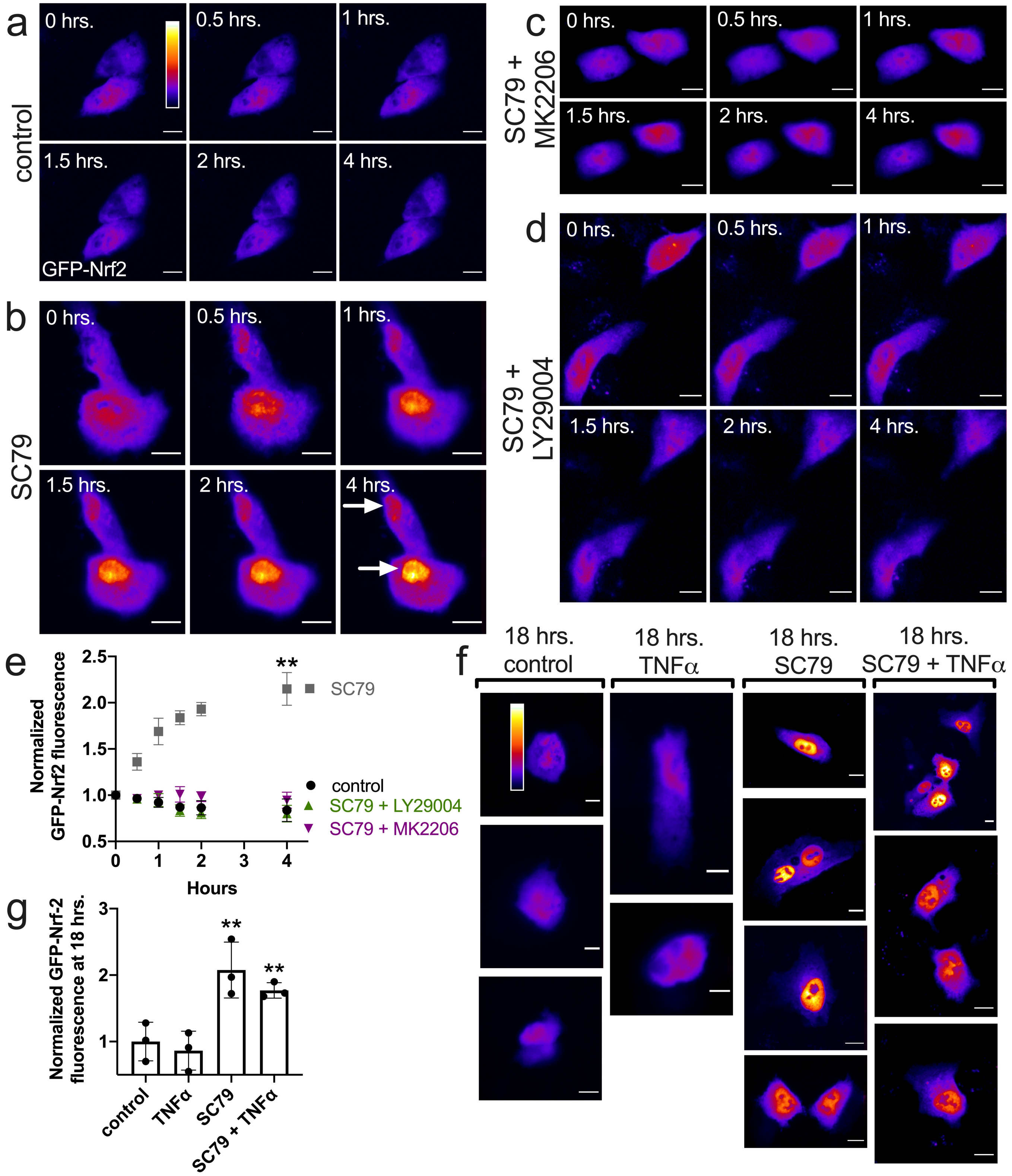
a
control
0 hrs.
0.5 hrs.
1 hrs.
1.5 hrs.
2 hrs.
4 hrs.
GFP-Nrf2
b
SC79
0 hrs.
0.5 hrs.
1 hrs.
1.5 hrs.
2 hrs.
4 hrs.
c
SC79 + MK2206
0 hrs.
0.5 hrs.
1 hrs.
1.5 hrs.
2 hrs.
4 hrs.
d
SC79 + LY29004
0 hrs.
0.5 hrs.
1 hrs.
1.5 hrs.
2 hrs.
4 hrs.
e
Normalized GFP-Nrf2 fluorescence
Hours
**
SC79
control
SC79 + LY29004
SC79 + MK2206
f
18 hrs. control
18 hrs. TNFα
18 hrs. SC79
18 hrs. SC79 + TNFα
g
Normalized GFP-Nrf-2 fluorescence at 18 hrs.
**
**
control
TNFα
SC79
SC79 + TNFα

Figure S10. SC79 induces nuclear GFP-Nrf-2. A549s on 8-well chambered coverglass were transfected with GFP-Nrf-2 (Addgene # 21549; (26)) using 0.05 µg GFP-Nrf-2 DNA + 0.25 µg empty pcDNA3.0 vector per well to limit GFP-Nrf-2 overexpression. Higher levels of GFP-Nrf-2 cDNA lead to nuclear accumulation at baseline. **(a-d)** Living cells were imaged every 30 min over the course of 2 hours then once again at 4 hours. A programable motorized stage (Prior Scientific, Rockland MA) was used on an inverted Olympus (Tokyo, Japan) IX83 microscope with 40x (0.75 NA) objective and standard FITC filter set. Unstimulated cells (*a*) were compared with cells stimulated with SC79 (1 µg/ml; *b*) or SC79 + 5 µM Akt inhibitor MK2206 (*c*) or SC79 + 1 µM PI3K inhibitor LY29004 (*d*). Inhibitors were added to cells 30 min prior to the experiment. SC79 was added at time 0. Images shown are from 0, 0.5, 1, 1.5, 2, and 4 hours after stimulation. Not increase in bright nuclear fluorescence with SC79 alone. **(e)** Results were quantified by taking the total GFP-Nrf-2 cellular fluorescence (normalized to time 0) from 3 different experiments, each experiment imaging 2-8 GFP-Nrf-2 transfected cells. Significance at 4 hours was determined by one-way ANOVA with Dunnett's posttest comparing all values to unstimulated control. Only SC79 alone was significantly different from control; ** $p<0.01$. **(f)** In separate experiments, cells were imaged at 18 hours after no stimulation (control) or 18 hours 1 µg/ml SC79 and/or TNF-α. Note strong nuclear fluorescence in cells with SC79 vs control or TNF-α. Images are shown from one single experiment per condition, each experiment imaging 3 independent random fields. Images are representative of 3 experiments per condition. Together with immunofluorescence of endogenous Nrf-2 in the Figure S9, these experiments support that SC79 activation of Akt enhances nuclear Nrf-2 levels under unstimulated and TNF-α stimulated conditions.

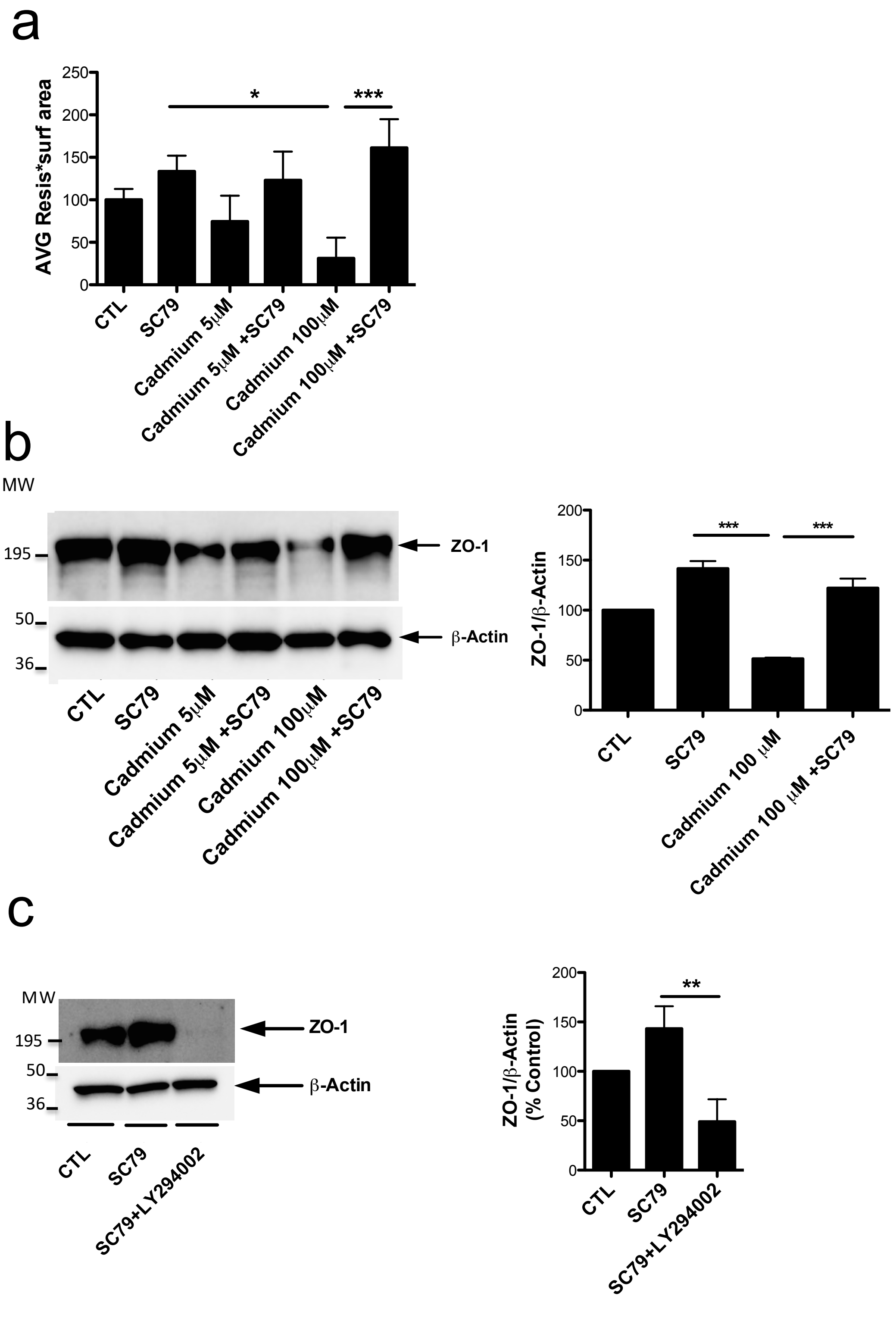
a
AVG Resis*surf area
250
200
150
100
50
0
*
***
CTL
SC79
Cadmium 5μM
Cadmium 5μM +SC79
Cadmium 100μM
Cadmium 100μM +SC79
b
MW
195
50
36
ZO-1
β-Actin
CTL
SC79
Cadmium 5μM
Cadmium 5μM +SC79
Cadmium 100μM
Cadmium 100μM +SC79
ZO-1/β-Actin
200
150
100
50
0
***
***
CTL
SC79
Cadmium 100 μM
Cadmium 100 μM +SC79
c
MW
195
50
36
ZO-1
β-Actin
CTL
SC79
SC79+LY294002
ZO-1/β-Actin
(% Control)
200
150
100
50
0
**
CTL
SC79
SC79+LY294002

Figure S11. Akt activation protects airway cells against cadmium-induced airway barrier dysfunction. **(a)** 16HBE14o- cells were grown in ALI-cultures and were treated with cadmium ± SC79 for 24 h and TEER measurements were taken. Bars are means ± SEM; n=3 experiments. **p*<0.05 (SC vs. cadmium 100μM) and ***p<0.001 (cadmium 100μM vs. cadmium +SC79) by ANOVA and Bonferroni posttest. **(b)** Cells were treated as in Fig. S8a and cell lysates were collected for Western blotting to detect ZO-1. Bars are means ± SEM; n=3 experiments. ****p*<0.001 (SC vs. cadmium) and ****p*<0.001 (cadmium 100μM + SC) by ANOVA and Bonferroni posttest. **(c)** Cells were treated with SC79 ± PI3K inhibitor (LY294002,10μg/ml) for 2 h and cell lysates were collected for Western blotting to detect ZO-1. Bars are means ± SEM; n=3 experiments. ***p*<0.001 (SC vs. SC+LY) by ANOVA and Bonferroni posttest.

Additional References

1. Hariri BM, McMahon DB, Chen B, Freund JR, Mansfield CJ, Doghramji LJ, et al. Flavones modulate respiratory epithelial innate immunity: anti-inflammatory effects and activation of the T2R14 receptor. J Biol Chem. 2017;292(20):8484-97.

2. Freund JR, Mansfield CJ, Doghramji LJ, Adappa ND, Palmer JN, Kennedy DW, et al. Activation of airway epithelial bitter taste receptors by Pseudomonas aeruginosa quinolones modulates calcium, cyclic-AMP, and nitric oxide signaling. J Biol Chem. 2018;293(25):9824-40.

3. Gruenert DC, Willems M, Cassiman JJ, Frizzell RA. Established cell lines used in cystic fibrosis research. J Cyst Fibros. 2004;3 Suppl 2:191-6.

4. Leino MS, Loxham M, Blume C, Swindle EJ, Jayasekera NP, Dennison PW, et al. Barrier disrupting effects of alternaria alternata extract on bronchial epithelium from asthmatic donors. PLoS One. 2013;8(8):e71278.

5. Winton HL, Wan H, Cannell MB, Gruenert DC, Thompson PJ, Garrod DR, et al. Cell lines of pulmonary and non-pulmonary origin as tools to study the effects of house dust mite proteinases on the regulation of epithelial permeability. Clin Exp Allergy. 1998;28(10):1273-85.

6. Shaul PW, North AJ, Wu LC, Wells LB, Brannon TS, Lau KS, et al. Endothelial nitric oxide synthase is expressed in cultured human bronchiolar epithelium. J Clin Invest. 1994;94(6):2231-6.

7. German Z, Chambliss KL, Pace MC, Arnet UA, Lowenstein CJ, Shaul PW. Molecular basis of cell-specific endothelial nitric-oxide synthase expression in airway epithelium. J Biol Chem. 2000;275(11):8183-9.

8. Kirsch EA, Yuhanna IS, Chen Z, German Z, Sherman TS, Shaul PW. Estrogen acutely stimulates endothelial nitric oxide synthase in H441 human airway epithelial cells. Am J Respir Cell Mol Biol. 1999;20(4):658-66.

9. Gopallawa I, Freund JR, Lee RJ. Bitter taste receptors stimulate phagocytosis in human macrophages through calcium, nitric oxide, and cyclic-GMP signaling. Cell Mol Life Sci. 2020.

10. Gao X, Zhang J. Spatiotemporal analysis of differential Akt regulation in plasma membrane microdomains. Mol Biol Cell. 2008;19(10):4366-73.

11. Vincent EE, Elder DJ, Curwen J, Kilgour E, Hers I, Tavare JM. Targeting non-small cell lung cancer cells by dual inhibition of the insulin receptor and the insulin-like growth factor-1 receptor. PLoS One. 2013;8(6):e66963.

12. Zhao Y, Hu X, Liu Y, Dong S, Wen Z, He W, et al. ROS signaling under metabolic stress: cross-talk between AMPK and AKT pathway. Mol Cancer. 2017;16(1):79.

13. Valentine RJ, Coughlan KA, Ruderman NB, Saha AK. Insulin inhibits AMPK activity and phosphorylates AMPK Ser(4)(8)(5)/(4)(9)(1) through Akt in hepatocytes, myotubes and incubated rat skeletal muscle. Arch Biochem Biophys. 2014;562:62-9.

14. Hawley SA, Ross FA, Gowans GJ, Tibarewal P, Leslie NR, Hardie DG. Phosphorylation by Akt within the ST loop of AMPK-alpha1 down-regulates its activation in tumour cells. Biochem J. 2014;459(2):275-87.

15. Ning J, Xi G, Clemmons DR. Suppression of AMPK activation via S485 phosphorylation by IGF-I during hyperglycemia is mediated by AKT activation in vascular smooth muscle cells. Endocrinology. 2011;152(8):3143-54.

16. Sample V, Ramamurthy S, Gorshkov K, Ronnett GV, Zhang J. Polarized activities of AMPK and BRSK in primary hippocampal neurons. Mol Biol Cell. 2015;26(10):1935-46.

17. Sundararaman A, Amirtham U, Rangarajan A. Calcium-Oxidant Signaling Network Regulates AMP-activated Protein Kinase (AMPK) Activation upon Matrix Deprivation. J Biol Chem. 2016;291(28):14410-29.

18. Condon KJ, Sabatini DM. Nutrient regulation of mTORC1 at a glance. J Cell Sci. 2019;132(21).

19. Dibble CC, Cantley LC. Regulation of mTORC1 by PI3K signaling. Trends Cell Biol. 2015;25(9):545-55.

20. Manning BD, Toker A. AKT/PKB Signaling: Navigating the Network. Cell. 2017;169(3):381-405.

21. Zhou X, Clister TL, Lowry PR, Seldin MM, Wong GW, Zhang J. Dynamic Visualization of mTORC1 Activity in Living Cells. Cell Rep. 2015.

22. Zhou X, Li S, Zhang J. Tracking the Activity of mTORC1 in Living Cells Using Genetically Encoded FRET-based Biosensor TORCAR. Curr Protoc Chem Biol. 2016;8(4):225-33.

23. Zhou BP, Hu MC, Miller SA, Yu Z, Xia W, Lin SY, et al. HER-2/neu blocks tumor necrosis factor-induced apoptosis via the Akt/NF-kappaB pathway. J Biol Chem. 2000;275(11):8027-31.

24. Zeng M, van der Donk WA, Chen J. Lanthionine synthetase C-like protein 2 (LanCL2) is a novel regulator of Akt. Mol Biol Cell. 2014;25(24):3954-61.

25. Xu Y, Duan C, Kuang Z, Hao Y, Jeffries JL, Lau GW. Pseudomonas aeruginosa pyocyanin activates NRF2-ARE-mediated transcriptional response via the ROS-EGFR-PI3K-AKT/MEK-ERK MAP kinase signaling in pulmonary epithelial cells. PLoS One. 2013;8(8):e72528.

26. Furukawa M, Xiong Y. BTB protein Keap1 targets antioxidant transcription factor Nrf2 for ubiquitination by the Cullin 3-Roc1 ligase. Mol Cell Biol. 2005;25(1):162-71.
